# Diffusional Voltammetry in Finite Spaces

**DOI:** 10.1021/acselectrochem.5c00091

**Published:** 2025-06-02

**Authors:** Yoshua H. Moore, Ben A. Johnson, Nicolas Plumeré

**Affiliations:** 9184 Technical University of Munich (TUM), Campus Straubing for Biotechnology and Sustainability, Uferstraße 53, 94315 Straubing, Germany

**Keywords:** Diffusional voltammetry, Porous electrodes, Pore structure characterization, Finite diffusion space

## Abstract

The pore structure is a key design parameter for optimizing electrocatalytic systems that utilize porous electrodes, necessitating characterization at scales relevant to catalysis (∼0.1–100 μm). In this Review, we examine how diffusion during faradaic processes is impacted by the electrode pore geometry, defined by the concavity/convexity of its surface curvature, and by pore size, defined by the finiteness of the diffusion domain. We briefly outline experimental considerations for correlating experimental and simulated data from porous electrodes, and then outline the current theories for modeling diffusional voltammetry at various electrodes with finite diffusion spaces (direct problem), including planar redox-active films, concave inverse opals and hollow tubes, and convex pillar arrays and particle arrays. Finally, we describe how these theoretical frameworks can be applied to characterize the electrode pore structure by analyzing experimental voltammetric current responses (inverse problem).

## Graphical abstract

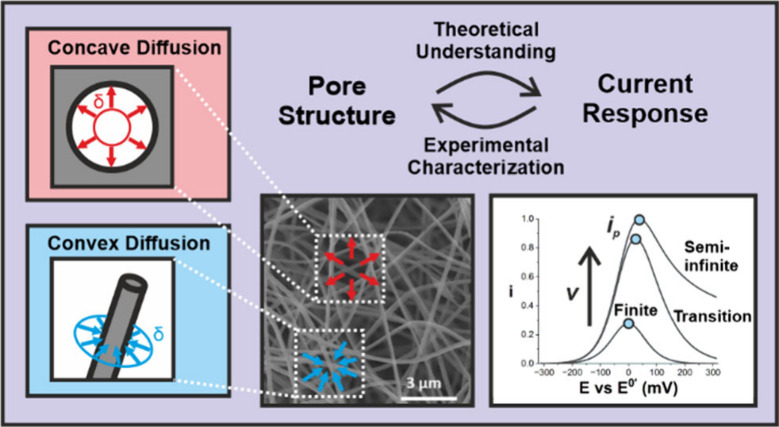

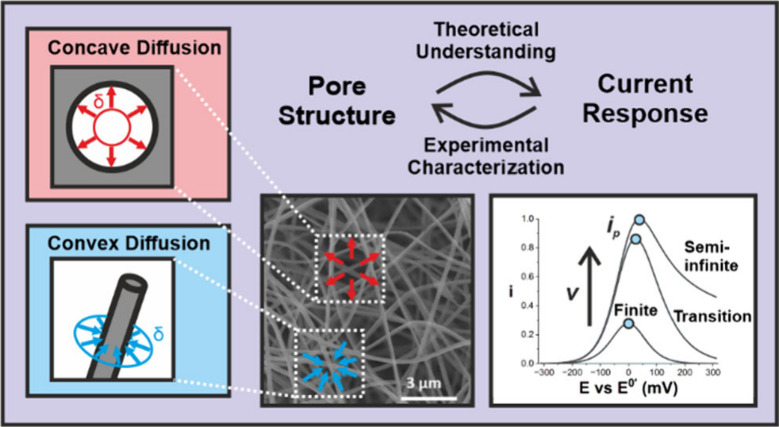

## Introduction

Porous electrodes are essential for advancing energy conversion systems toward commercial viability, as they enable the high conversion rates necessary for practical applications. This performance enhancement arises from the increased catalyst loading per unit area of the electrode footprint and the enhanced mass transport of reactants and products facilitated by the porous structure.^1–3^ The structure of porous electrodes strongly influences key performance metrics such as faradaic efficiency,^1,4–8^ product selectivity^1,6,9^ and current density.^4–10^ Moreover, incorporating porosity into models of porous electrodes is paramount for accurately simulating electrochemical systems^11–13^ and guiding bottom-up experimental designs.^3,14,15^ Consequently, precise characterization of the pores is critical to optimizing electrode performance.

A tailored methodology is needed to characterize pore size and geometry on scales relevant to catalysis. For experimental time windows between ∼20 μs and 200 s^16^ and typical diffusion coefficients around 10^–5^ cm^2^/s, the corresponding diffusion length scales are on the order of 0.1–100 μm. At these scales, features such as microporosity, nanoroughness, or surface areas derived from capacitive measurements may be less relevant. Furthermore, methods such as electron microscopy imaging cannot characterize features in the bulk pore structure.

A key aspect of diffusional voltammetry is the interplay between diffusional mass transport, electron transfer kinetics, and ohmic drop, which shape the voltammetric current response. By leveraging diffusion and surface reactions of redox probes within pores that constrain diffusion to a maximum finite distance, insights into the pore size, distribution, and geometry of the electroactive bulk pore on scales that directly impact catalytic performance may be elucidated. This can be achieved using electrochemical modeling to understand how pore structure affects diffusion-controlled voltammetric current signals (direct problem), and then using this knowledge to correlate experimental data with simulations in order to extract structural information (inverse problem). We focus on macropores on scales ranging from 0.1 to 100 μm, which are large enough for systems to behave as fully-supported to minimize migration^17^ by assuming infinite dilution, electroneutrality, and absence of potential gradients. Additionally, we will consider the systems as stationary to mitigate convection, leaving diffusion as the sole transport mechanism. While surface roughness of electrodes can produce similar effects to porosity in shaping voltammetric responses,^18^ this aspect lies outside the scope of this Review. We distinguish porosity from roughness based on how the dimensionality of the surface structure affects diffusion in the long time scale domain: roughness features (2D topography) lead to semi-infinite diffusion, whereas porosity features (3D topography) lead to finite diffusion.

Here, we introduce how pore structure affects diffusion dynamics within pores. This is followed by a brief discussion of the experimental challenges involved in collecting and interpreting voltammetric data from porous electrodes. Finally, we review the theory of diffusional voltammetry at electrodes with finite diffusion spaces, providing insights for applying this theoretical framework to elucidate pore structure information on electrode samples based on their voltammetric current outputs.

## The Influence of Pore Structure on Diffusion

Net diffusion occurs when a species moves from regions of high concentration to low concentration, driven by random molecular Brownian motion. This process is governed by the diffusion equation (eq 1), which describes how concentration evolves over time and space. The accumulation term $\frac{\partial C}{\partial t}$ represents the rate of change of concentration, and the flux term *D*∇^2^ *C* represents the net flux of the diffusing species, driven by spatial concentration gradients.

$$\frac{\partial C}{\partial t}=D\nabla^{2}C$$ 1

In electrochemistry, concentration changes primarily occur near the electrode-solution interface, where electrochemical reactions convert a substrate into a product. This creates a concentration gradient at the electrode, forming a diffusion layer: a region near the electrode surface where the concentration differs from its initial bulk value (*C* ^0^). The characteristic thickness of this region is referred to as the diffusion layer thickness (*δ*).

The diffusion layer thickness is dependent on:

1. The rate of conversion from substrate to product, which depends on the potential at the electrode relative to the formal potential of the redox probe (*E* – *E* ^0^′), and the kinetic rate constant of the heterogenous electron transfer (*k* ^0^).
2. The rate of diffusional flux governed by Fick’s First Law (*J* = *D*∇*C*) that depends on the diffusion coefficient (*D*) and the concentration gradient of the redox species (∇*C*).
3. The experimental time scale (*t*_*c*_) allowed for *δ* to expand.

In general, it is not possible to define an exact location where the diffusion layer ends and the bulk solution begins. As such, the exact value of the transient *δ* may include a multiplicative factor depending on how the boundary of the region over which substrates and products diffuse is approximated from the concentration profile. However, under the assumption of semi-infinite planar diffusion, *δ* can be asymptotically expressed as

$$\delta ∼\sqrt{Dt_{c}}$$ 2

In linear sweep voltammetry, the experiment time *t*_*c*_ is governed by the potential scan rate (*ν*)­

$$t_{c}=\frac{RT}{Fv}$$ 3

with (*R*) the molar gas constant, (*T*) the temperature, (*F*) Faraday’s constant.

This leads to a new expression for the diffusion layer thickness

$$\delta ∼{\left(\frac{RTD}{Fv}\right)}^{1/2}$$ 4

Importantly, the geometry of the electrode and the finiteness of the diffusion space affect the current response in electrochemical measurements.

### Pore Geometry: Surface Curvature

For plane curves, curvature is defined as the rate at which the angle (*ϑ*) of its tangent changes from point to point as one moves along its arc length (*s*), represented as *dϑ*/*ds*. This quantifies how sharply the curve bends at any given point. The curvature at a specific point can be described by an *osculating circle*–from the Latin *osculum*, meaning “to kiss”, which shares both the tangent and the bending direction at that point. For a circle of radius *r*, curvature is defined as 1/*r*. As a curve straightens, a circle with larger radius is needed to approximate it, resulting in a lower curvature value.

For surfaces, the normal at any point along a surface defines a tangent plane (Figure 1) that can rotate along two perpendicular axes, one vertical and one horizontal. The two axes result in two *principal curvatures* (*K*_1_) and (*K*_2_) that are thus also perpendicular to each other.

**Figure 1:**
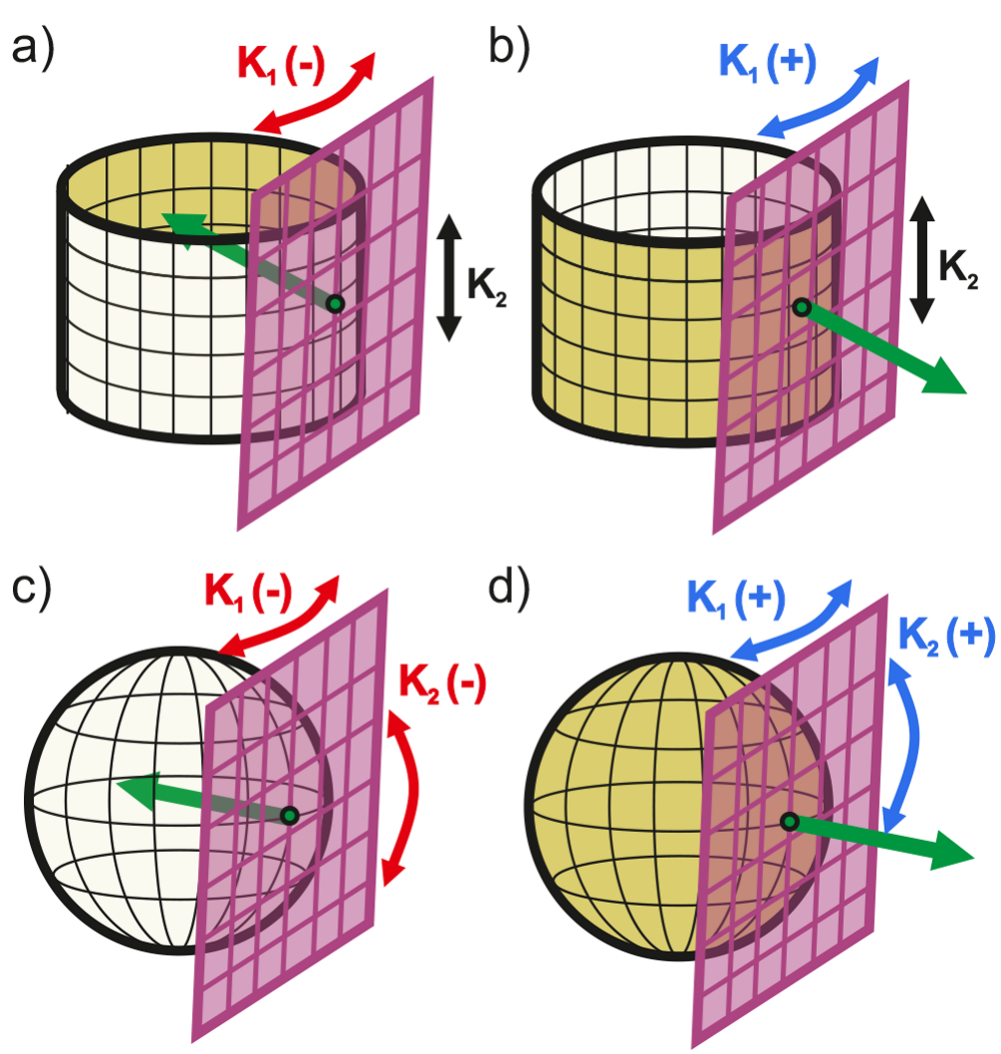
Schematic illustrating the surface normal (green arrows) and tangent planes (pink) on curved surfaces for (a) a concave cylinder, (b) a convex cylinder, (c) a concave sphere, and (d) a convex sphere. The rotations of the tangent plane in both vertical and horizontal directions represent the principal curvatures at each point, resulting in the mean curvature. Negative (−), zero, and positive (+) curvatures are indicated in red, black, and blue, respectively.

The sign of each principal curvature is determined by the orientation of the surface relative to the normal. If the surface curves in the same direction as the normal vector (Figure 1a and c), the curvature is considered negative (concave). Conversely, if the surface curves in the opposite direction of the normal vector (Figure 1b and d), the curvature is positive (convex).

The mean curvature (*H*) at this point is the average sum of the two principal curvatures (eq 5). For example, the concave cylinder of radius *r* has principal curvatures of *K*_1_ = –1/*r* (circular cross section) and *K*_2_ = 0 (flat length), resulting in *H* = –1/2*r*. A convex sphere of radius *r* has principal curvatures of *K*_1_ = 1/*r* and *K*_2_ = 1/*r*, resulting in *H* = 1/*r*.

$$H=\frac{1}{2}\left(K_{1}+K_{2}\right)$$ 5

In diffusional voltammetry, the mean curvature *H* of the pore surface (analogous to the pore geometry) affects the direction of the diffusional flux towards it, which in turn affects how the diffusion layer progresses over time. This direction can be described in terms of the concavity or convexity of the pore (Figure 2).

**Figure 2:**
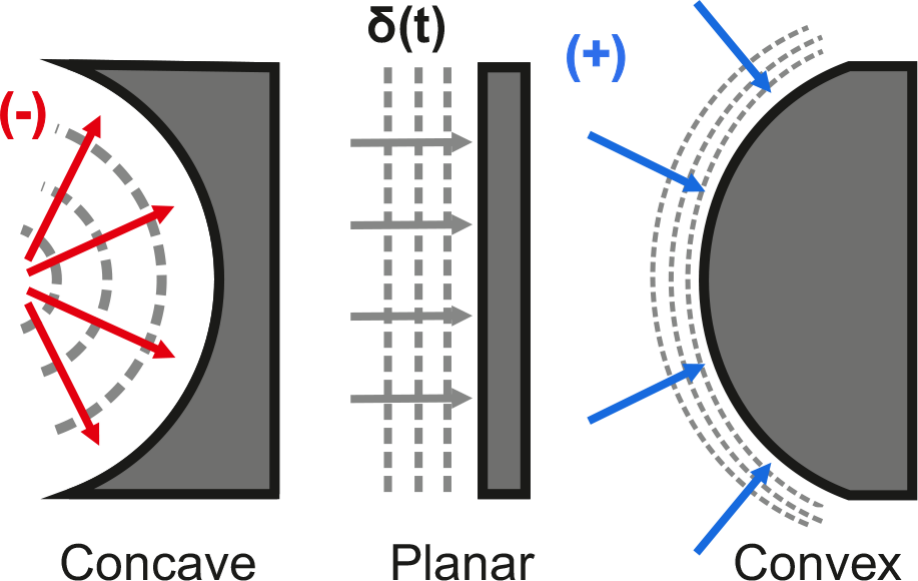
Schematic showing the expansion of the diffusion layer over time for concave, planar, and convex electrode surfaces.

For concave geometries, the diffusion layer boundary gets smaller as it progresses over time (analogous to inverted hemispherical diffusion). For planar geometries, the diffusion layer boundary remains the same as it progresses over time (analogous to linear diffusion). For convex geometries, the diffusion layer boundary gets larger as it progresses over time (analogous to hemispherical diffusion).

Examples of electrode geometries that display diffusion dynamics ranging from concave to convex include surfaces shaped as an inverse opal, hollow cylinder, plane, pillar and particle, as shown in Figure 3a-e, respectively.

**Figure 3:**
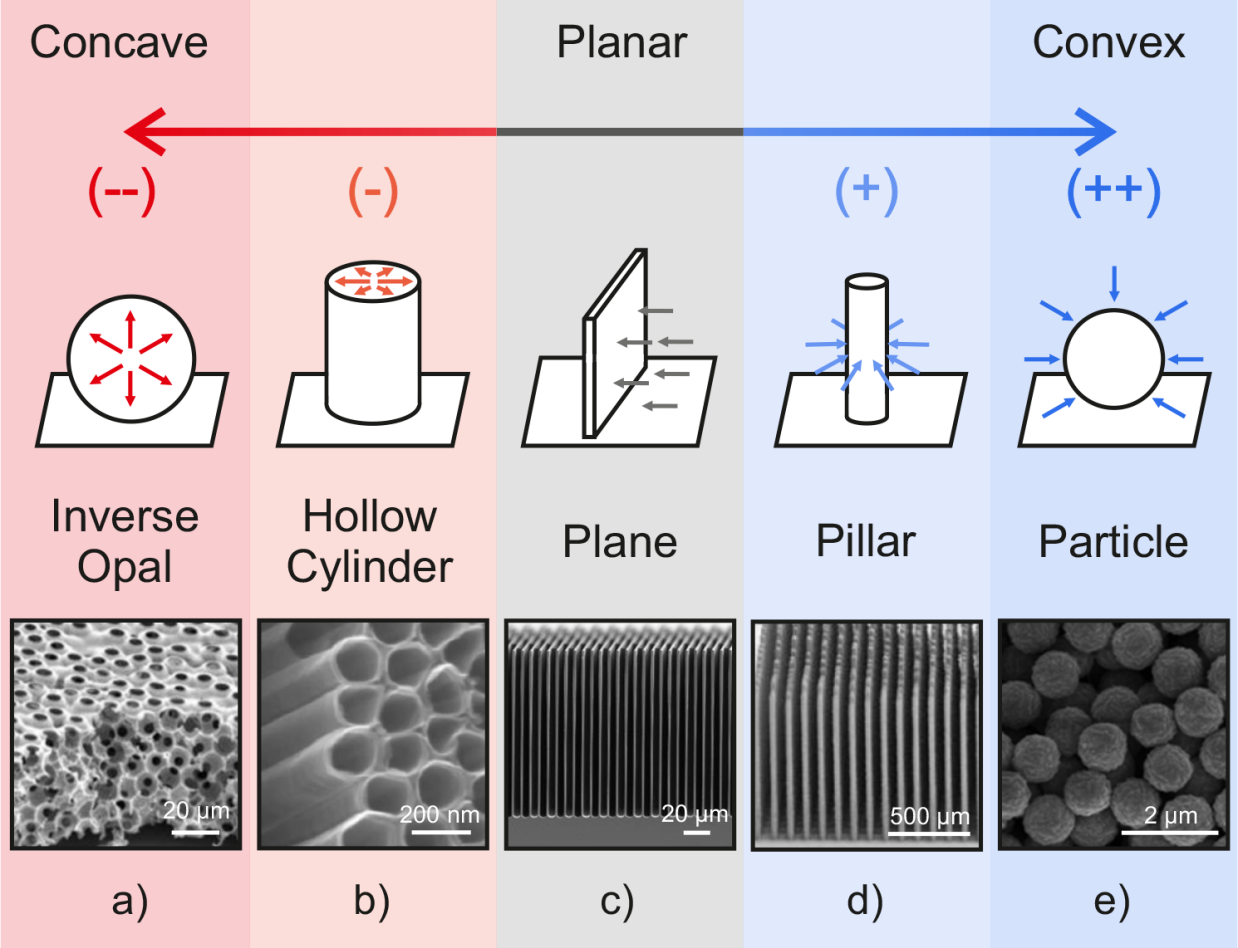
Schematic illustrating various pore geometries that lead to concave (red), intermediate planar (gray), or convex (blue) diffusion, as well as SEM image examples of electrodes for each geometry: (a) ITO inverse opals, (b) TiO_2_ nanotubes, (c) TiO_2_ trenches, (d) ITO pillars, and (e) Cu_2_O particles. Panel (a) reprinted from ref 19. Copyright 2018 American Chemical Society. Panel (b) reprinted with permission from ref 20. Copyright 2013 The Royal Society of Chemistry. Panel (c) reprinted with permission from ref 21 under CC BY 4.0. Panel (d) reprinted with permission from ref 9. Copyright 2022 Springer Nature BV. Panel (e) reprinted with permission from ref 22 under CC BY 4.0.

### Pore Size: Finiteness of the Domain

Porous electrodes have finite domains, which occurs when the diffusion volume is constrained by a physical boundary that prevents the diffusion layer from expanding beyond a maximum distance (*δ*_*max*_) that we define as the characteristic geometric length scale ( l ). Note that, for a convex geometry to display finite diffusion characteristics, the electrode must be configured as an array such that neighboring diffusion layers overlap.

For example, three porous electrodes with a concave, planar and convex pore geometry that exhibit confined diffusion are inverse opal, redox-active films, which exhibit apparent diffusion via electron hopping between redox moieties,^23^ and pillar array electrodes, respectively. The characteristic length l for each pore geometry is shown in Figure 4a, which for redox-active films is the film thickness (*d*), for inverse opals is the pore radius (*R*_*pore*_), and for pillar arrays is the midpoint distance between two pillars (*d*_*interpillar*_).

**Figure 4:**
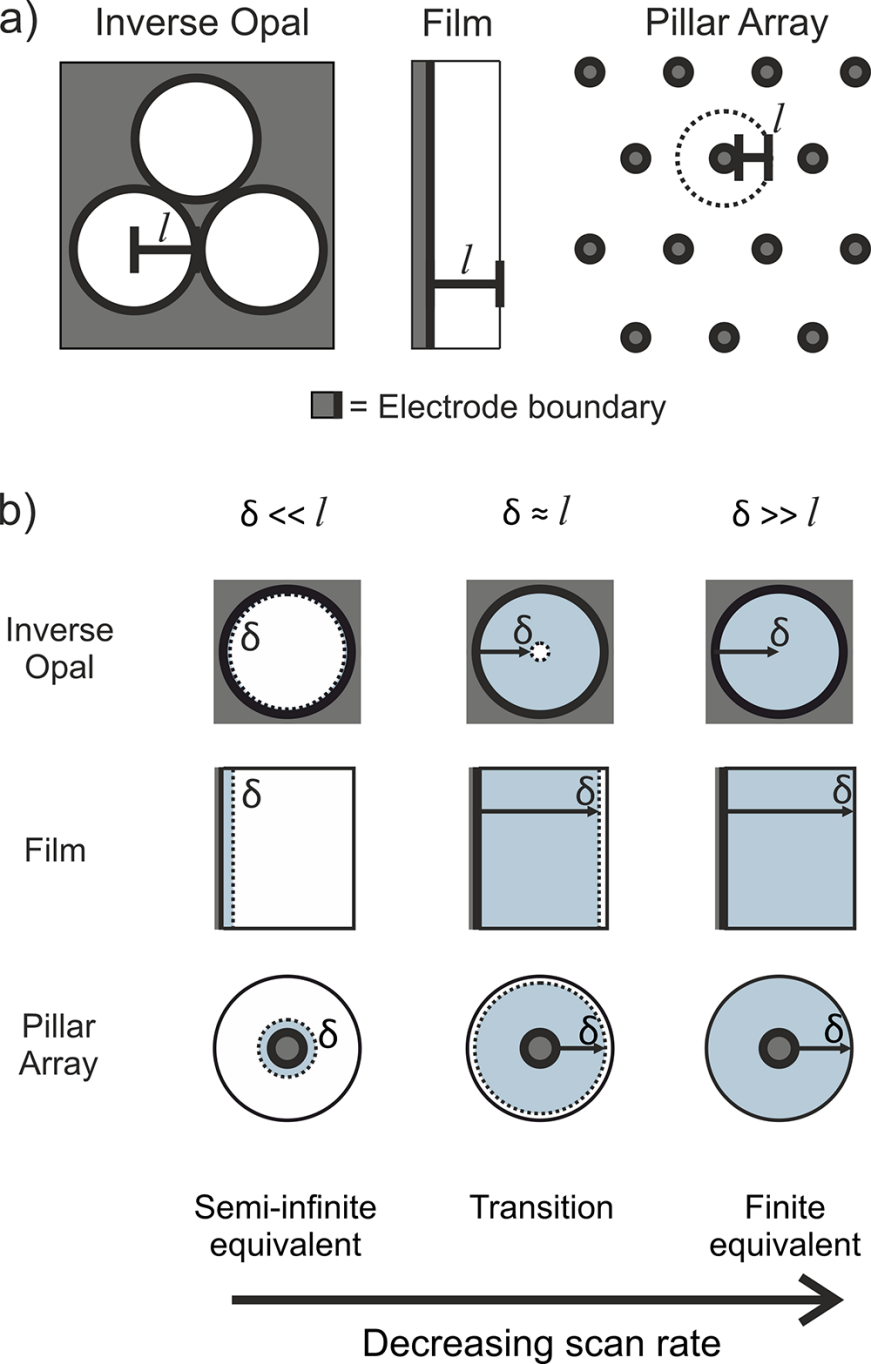
Schematics of (a) the characteristic length ( l ) that defines the finite domain for an inverse opal, film, and pillar array electrode (for pillar arrays, the hexagonal base of the diffusion domain is converted into a circular equivalent) and (b) the diffusion regimes for these geometries as the scan rate decreases.

The “finiteness” of the diffusion space can be defined as the ratio of l to *δ*, which leads to the dimensionless confinement parameter (*w* ^1/2^). Expanded for voltammetry, this results in

$$w^{1/2}=\frac{l}{\delta }=\frac{lv^{1/2}}{D^{1/2}{\left(RT/F\right)}^{1/2}}$$ 6

From eq 6 it follows that selecting different scan rates directly affects the diffusion layer thickness *δ*, providing an experimental handle to probe different length scales with respect to l . Three characteristic confinement regimes arise depending on the value of *w* ^1/2^, illustrated schematically in Figure 4b.

- At low *w* ^1/2^ (*δ* ≫ l ), a finite regime occurs, where all the substrate within the diffusion volume reacts at the electrode surface.
- At high *w* ^1/2^ (*δ* ≪ l ), a semi-infinite regime occurs, where only a small fraction of the substrate reacts, leaving most of the diffusion volume unperturbed.
- At intermediate *w* ^1/2^ (*δ* ≈ l ), a transition regime occurs, where the diffusion behavior gradually shifts from finite to semi-infinite.

### Semi-quantitatively Relating Diffusion Dynamics to Pore Structure

Inspired by Kac’s seminal work^24^ *Can One Hear the Shape of a Drum?*, Kant demonstrated that key morphological information, such as curvature and correlation length (the distance between peaks in surface roughness), of a randomly rough electrode influences the diffusion-limited current transient, thus setting the foundation for electrode characterization.^25,26^ An analytical approach for extracting the statistical morphology information from rough electrodes was proposed along with exploring its experimental feasibility.^27–29^ Here, we extend this demonstration from roughness to porosity (i.e., finiteness), by semi-quantitatively relating diffusion dynamics to various pore geometries with different surface curvatures.

The time scale at which the diffusion layer reaches the outer boundary of the finite domain depends on both its geometry and size. Consequently, monitoring the current response as a function of experiment time can be used to extract information about the geometry and size of a porous electrode. In general, for any geometry there exists a characteristic time scale defined as the point when the diffusion layer thickness is on the order of the critical geometric length scale of the electrode l , analogous to when *w* ^1/2^ ∼ 1 (eq 6). The time scale is given by

$$\tau =\sigma \left(\frac{l^{2}}{D}\right)$$ 7

where *σ* is a geometry-dependent constant. By evaluating *τ*, we can obtain semi-quantitative information not only on the size ( l ) of the finite domain but its geometry (*σ*) as well.

To demonstrate this principle, we consider the simplest case of diffusion within the five idealized geometric solids highlighted in Figure 3. The geometries can be described by an axisymmetric one-dimensional coordinate system, where a general form of the diffusion equation (eq 1) can be written as

$$\frac{\partial C}{\partial t}=D\frac{1}{x^{d-1}}\frac{\partial }{\partial x}\left(x^{d-1}\frac{\partial C}{\partial x}\right)$$ 8

where *d* is the *dimensionality*: *d* = 1 corresponds to planar coordinates, *d* = 2 to cylindrical coordinates, and *d* = 3 to spherical coordinates.

As shown in Figure 5, depending on the position of an outer boundary of distance l relative to the electrode surface, the resulting diffusion profile will be concave or convex. We set an equivalent value of l for each geometry to compare geometry-effects on *τ*. Consequently, we derived a general expression for the concentration profiles *C*(*x*, *t*) and current response *i*(*t*) in these three coordinate systems encompassing both convex and concave diffusion, enabling us to calculate *τ* for the five different geometries (see Supporting Information for the full derivation).

**Figure 5:**
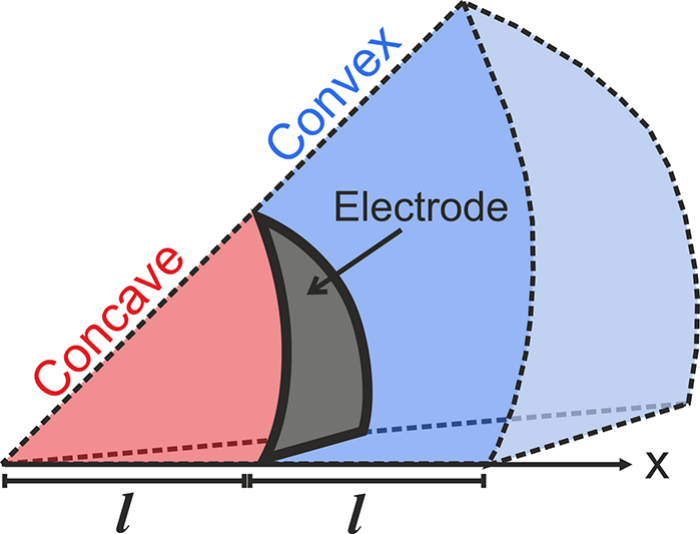
Schematic section of a spherically curved electrode boundary with a concave or convex finite volume, where l is the critical geometric length scale.

If a large potential step is applied, such that the concentration of a freely diffusing species is immediately reduced to zero at the electrode surface, a general solution to this problem can be found by an eigenfunction expansion. By expressing the concentration of the diffusing species *C*(*x*, *t*) in terms of eigenfunctions of the Laplacian operator $\frac{1}{x^{d-1}}\frac{\partial }{\partial x}\left(x^{d-1}\frac{\partial }{\partial x}\right)$ , this allows the spatial dependence to be decoupled from the time dependence (separation of variables), where the latter follows simple exponential decay. Using this approach, the concentration profiles are expressed as

$$C\left(x,t\right)=C^{0}\underset{n}{\sum }A_{n}\;\exp\left(-{\lambda_{n}}^{2}t\frac{D}{l^{2}}\right)\phi_{n}\left(x\right)$$ 9

where *A*_*n*_ are coefficients determined by the boundary conditions, *λ*_*n*_ are the eigenvalues for a given geometry, and *ϕ*_*n*_(*x*) are the corresponding eigenfunctions. These three components are geometry-dependent; that is, they vary between planar, cylindrical, and spherical geometries as well as between concave or convex diffusion (see Supporting Information for mathematical justification and derivations). Furthermore, if the electrode is placed at *x* = l (Figure 5), the current response is given by

$$i=SFC^{0}\frac{D}{l}\underset{n}{\sum }A_{n}\;\exp\left(-{\lambda_{n}}^{2}t\frac{D}{l^{2}}\right)\frac{d\phi_{n}}{dx}\left(l\right)$$ 10

where (*S*) is the electrode surface area. The characteristic diffusion time *τ*, when the diffusion layer thickness is on the order of the characteristic geometric length scale, will correspond to the *longest time scale* in eq 9 and eq 10. By inspection, longest time scale in these expressions corresponds to the largest time constant in the exponential term, which coincides *with the smallest eigenvalue λ*_0_. Thus, to determine the geometry-dependence of *τ*, one only needs to examine the smallest eigenvalue of the Laplacian operator for a given geometry and coordinate system. By combining this exact result with eq 6, the characteristic diffusion time can be written as

$$\tau =\frac{1}{{\lambda_{0}}^{2}}\left(\frac{l^{2}}{D}\right)$$ 11

A list of characteristic diffusion times for the five idealized geometric solids is given in Table 1.

**Table 1:** List of Characteristic Diffusion Times τ and Their Approximate Values for Various Pore Geometries

	*τ*	*τ*/*τ*_plane_
Concave Sphere	$\frac{1}{\pi^{2}}\left(\frac{l^{2}}{D}\right)$	0.25
Concave Cylinder	$0.17\left(\frac{l^{2}}{D}\right)$	0.43
Plane	$\frac{4}{\pi^{2}}\left(\frac{l^{2}}{D}\right)$	1
Convex Cylinder	$0.54\left(\frac{l^{2}}{D}\right)$	1.33
Convex Sphere	$0.74\left(\frac{l^{2}}{D}\right)$	1.82

It is important to note that for any geometry, even complex structures and irregular pore networks, the diffusion layer thickness will always scale as $\delta ∼\sqrt{Dt}$ , and the diffusion time as *τ* ∼ l ^2^/*D*, where l is the smallest critical length scale. This can be deduced from the general mass conservation given in eq 1. What differs across geometries is a constant multiple, for example, *σ* in eq 7.

The ratio *τ*/*τ*_plane_ offers a semi-quantitative description of how electrode geometry influences diffusion dynamics, and by extension current outputs. Concave geometries with *τ*/*τ*_plane_ < 1 correspond to slower diffusion dynamics, flat geometries with *τ*/*τ*_plane_ = 1 correspond to planar diffusion, and convex geometries with *τ*/*τ*_plane_ > 1 correspond to faster diffusion dynamics. This highlights the possibility of determining electrode geometry from diffusional current output.

## Experimental Considerations for Correlating Experiments to Simulations

When correlating experimental data to simulations, it is important that experimental conditions satisfy the assumptions of the electrochemical model, namely, that the recorded current signals are strictly diffusion-controlled. This requires careful mitigation of effects such as ohmic drop^30^ and ion permeation,^31^ achievable through IR-feedback compensation, the use of a sufficient concentration of supporting electrolyte and counter ions, and an optimized electrode setup. Additionally, it has been reported that porous electrodes with hydrophobic surfaces, such as graphite, require a preliminary wetting step using a non-aqueous solvent^32^ to displace trapped air from within the pore and enable reproducible current measurements.

Moreover, correlating experimental data with simulations requires the determination of several key electrochemical parameters, which are used either for normalizing the experimental data or as input variables for simulations. These parameters typically include the electroactive surface area (*S*), the bulk concentration of the redox probe (*C* ^0^), the diffusion coefficient (*D*), and, for systems with non-reversible kinetics, the heterogeneous rate constant (*k* ^0^).

Determining *S*, *C* ^0^ and *D* is relatively straightforward using established methods.^16,32–36^ However, determining *k* ^0^ can be more challenging. The Nicholson method,^37^ often used to extract *k* ^0^ values from cyclic voltammetry, assumes planar diffusion, an assumption that may break down for porous electrodes.^38^ Therefore, using models that incorporate the appropriate geometry is recommended when fitting experimental and simulated peak positions. Measurements should also be taken at sufficiently high scan rates to approximate semi-infinite diffusion conditions. Notably, one study reported a distribution of *k* ^0^ values when examining individual fibers in a carbon felt electrode.^36^ In this case, an average *k* ^0^ value was insufficient to accurately fit cyclic voltammograms (CVs) across multiple scan rates, suggesting non-uniform kinetics throughout the electrode. Combining electrochemical impedance spectroscopy (EIS) and chronoamperometry (CA), have been proposed to overcome these challenges.^39^ Data processing can be greatly simplified by ensuring that systems exhibit reversible or near-reversible electron transfer kinetics, reducing the influence of inaccuracies in *k* ^0^ determination when later correlating experimental data to simulated currents.

## Voltammetry in Finite Spaces

For direct correlation and fitting between experimental and simulated current data, an electrochemical model should meet several key criteria:

1. A comprehensive system description: The model should fully describe the system, including the definition of relevant dimensionless groups related to pore characterization, and the treatment of heterogeneities.
2. Balanced geometric complexity: The model should realistically capture the complexity of the electrode geometry while balancing physical accuracy and computational efficiency. This introduces the concept of a *minimally-complex* model, where only the complexity essential to reproducing critical behavior is retained. Screening can be achieved by evaluating how changes in structural complexity affect the current output. For example, it was shown that a 3D model was necessary to correctly simulate nanowire electrodes, as a 2D model failed to capture important edge effects at the wire tips.^40^
3. Accessibility of simulated currents: Ideally, simulated current datasets should be readily accessible, rather than limited to correlation plots.
4. Experimental validation: Practical application of the model should be demonstrated through experimental studies, particularly in the context of solving the inverse problem (i.e., extracting pore structure information from voltammetric data).

In the remainder of this review, we highlight key studies that most closely meet these requirements across the five previously discussed pore geometries, covering structures with surface curvatures ranging from concave to convex.

### Homogeneous Planar Redox-Active Films

The simplest case of reversible voltammetry within a confined space subject to planar diffusion, applicable for redox-active films or thin layer cells, was described by Aoki and co-workers.^41^ Redox-active films consist of redox-active centers attached to a solvated supporting matrix (typically a polymeric material) immobilized on an electrode substrate, where electron hopping between adjacent redox-active centers is treated as diffusion of electrons.^23^ They examined the effect of the confinement parameter (*w*) on the features of a linear sweep voltammogram (LSV).

Their results showed that finite diffusion occurs when *w* ^1/2^ < 1.3, semi-infinite diffusion when *w* ^1/2^ > 6.9, and a transitionary regime exists between these values. These regimes appear as linear (Figure 6C), plateau (Figure 6A) and mixed regions (Figure 6B), respectively, when plotting the normalized peak current versus the normalized scan rate. They derived eq 12 to describe the peak current behavior across the entire range of *w* ^1/2^, allowing l to be determined assuming a uniform thickness of the finite space:

$$i_{p}=0.446FSC^{0}\frac{D}{l}w^{1/2}\;\tanh\left(0.56w^{1/2}+0.05w\right)$$ 12

**Figure 6:**
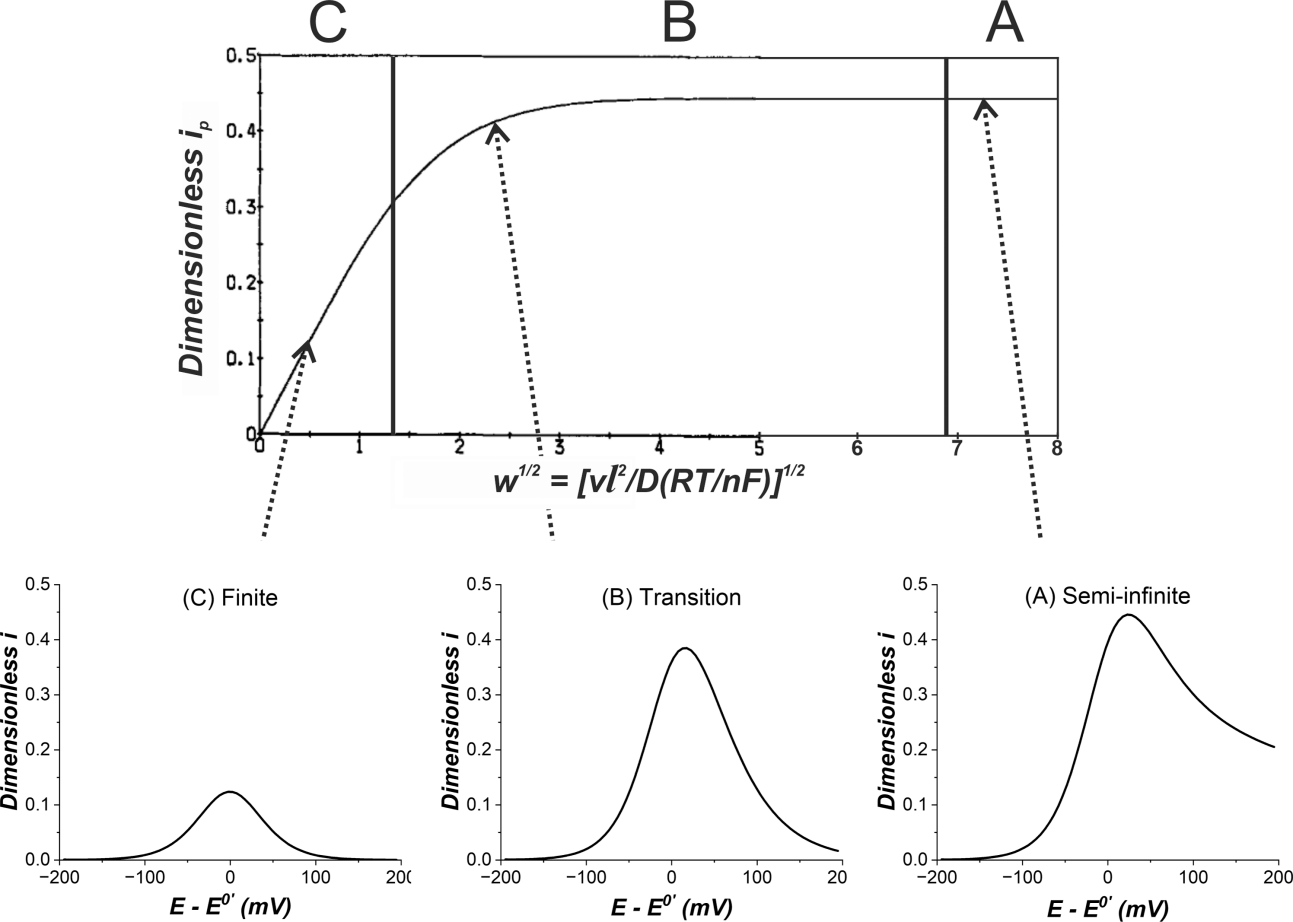
Variations of dimensionless peak currents with *w* ^1/2^. Regions A, B, and C denote semi-finite, transitionary, and finite diffusion. An example LSV for each regime is also shown. The dimensionless current (*i*) is defined in the original paper.^41^ Adapted with permission from ref 41. Copyright 1983 Elsevier.

The same system but for a non-reversible electron transfer process using the Butler-Volmer expression was examined in a subsequent paper.^42^ They investigated how electrode kinetics, represented by the dimensionless kinetic parameter Λ (eq 13), influence the features of an LSV as a function of confinement, where *k* ^0^ is the heterogeneous rate constant. The parameter Λ defines whether the electron transfer is reversible, quasi-reversible, or irreversible:

$$\Lambda =k^{0}{\left(\frac{RT}{FDv}\right)}^{1/2}$$ 13

Combination of the dimensionless kinetic parameter Λ with the dimensionless confinement parameter *w* yielded a new dimensionless parameter ψ (eq 14), which directly compares the rate of electron transfer at the electrode surface with the rate of diffusion towards it. Since ψ is independent of the scan rate, it serves as a descriptor of the intrinsic electrochemical kinetics of the system:

$$\psi =\Lambda w^{1/2}=\frac{k^{0}l}{D}$$ 14

They constructed a dimensionless plot of normalized peak currents *i*_*p*_ versus the confinement parameter *w* ^1/2^ for various values of ψ (Figure 7a) and provided a zone diagram (Figure 7b) indicating the range of *w* ^1/2^ values over which different diffusion regimes occur, based on Λ. For the irreversible case (region 3 on Figure 7b), they derived an approximate equation (eq 15), where *α* is the symmetry coefficient, that is valid across all *w*. This enables the determination of l for irreversible electron transfer kinetics, assuming a uniform thickness of the finite space:

$$i_{p}=0.496F{SC}^{0}{\left(\frac{\alpha FDv}{RT}\right)}^{1/2}\;\tanh\left(0.742{\left(\alpha w\right)}^{1/2}+0.015\alpha w\right)$$ 15

**Figure 7:**
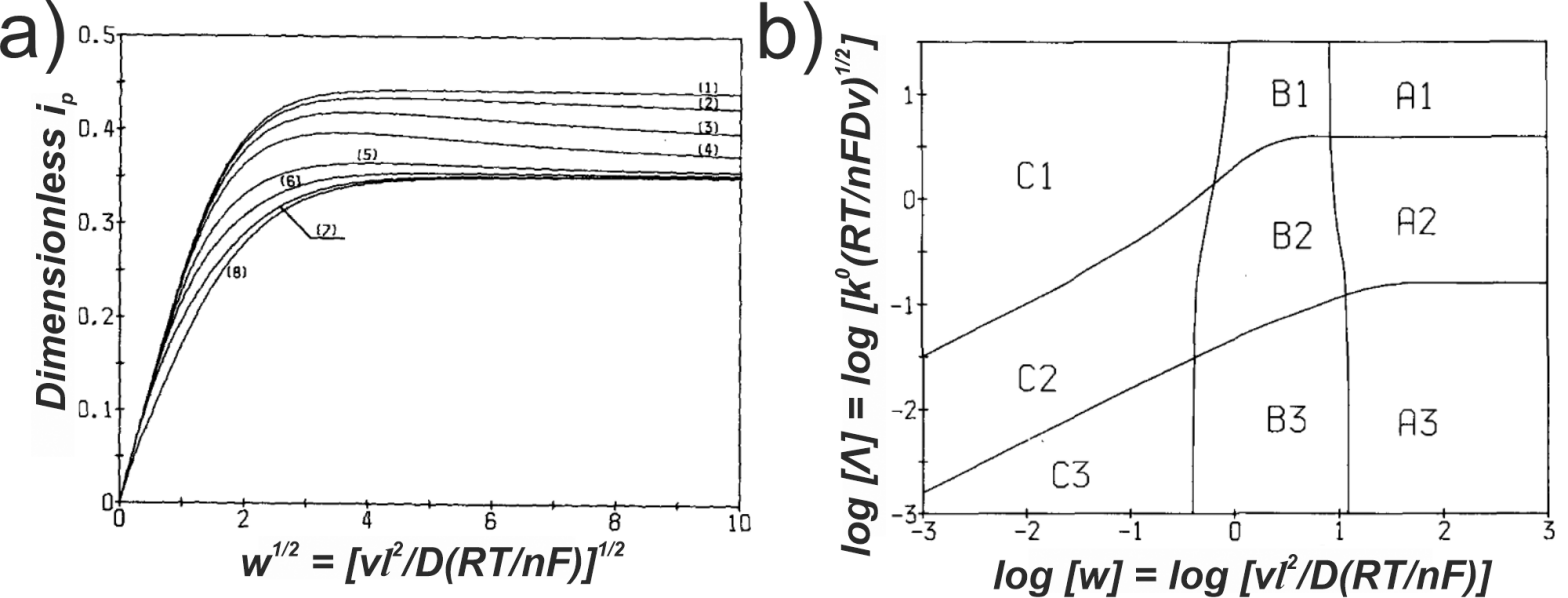
(a) Variations of the dimensionless peak current *i*_*p*_ versus scan rate *w* ^1/2^ for a series of ψ values: (1) 80, (2) 20, (3) 7, (4) 3, (5) 1, (6) 0.5, and (7) 0.01 when *α* = 0.5. (b) Domains of Λ and *w* for the degrees of reversibility and the finiteness of the diffusion space. 1, 2, and 3 denote the reversible, quasi-reversible, and irreversible kinetic regions, while A, B, and C denote semi-infinite, transitionary, and finite diffusion regimes, respectively. Panels (a, b) adapted with permission from ref 42. Copyright 1984 Elsevier.

### Heterogeneous Planar Redox-Active Films

Expansion on Aoki’s theoretical work for homogeneous finite planar systems (Figure 8a) to include film thickness heterogeneity (Figure 8b) was achieved by Buesen and co-workers.^43^ They stated that heterogeneity results in the entire film being represented by an average thickness ( l _*avg*_), which by extension also lead to an averaged confinement parameter (*w*_*avg*_):

$$w_{avg}=\frac{F{l_{avg}}^{2}v}{RTD}$$ 16

They showed how, in contrast to a homogenous (smooth) film (Figure 8a), where the diffusion regime is equivalent across the whole electrode surface for all time scales (*δ*_1_, *δ*_2_, *δ*_3_ for semi-finite, intermediate and finite, respectively), a heterogeneous (rough) film (Figure 8b) will have differing diffusion regimes at intermediate time scales depending on the value of l at different positions across the electrode surface. The net result is that the average normalized peak current response *i*_*p*_ will decrease for heterogeneous films at intermediate time scales *δ*_2_ (Figure 8c and d). The analysis of *i*_*p*_ allows for the extraction of the film thickness distribution of redox-active films by fitting simulations to experimental data.

**Figure 8:**
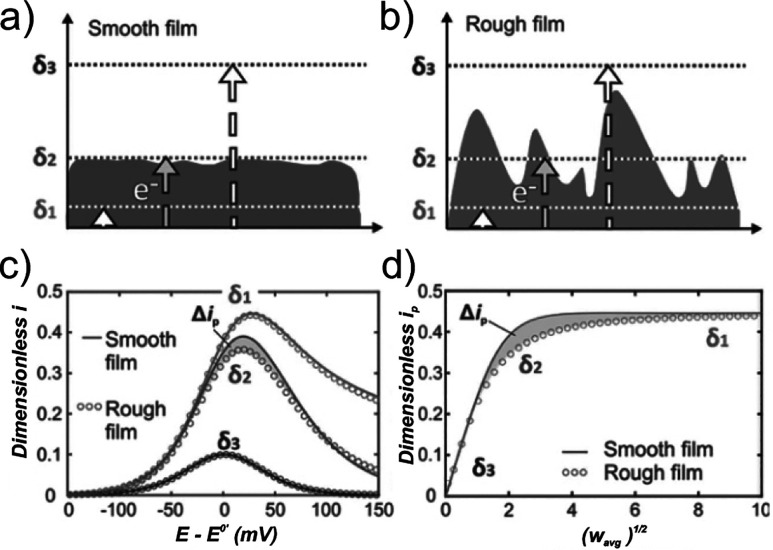
Schematic illustration of the diffusion layers of the electron (*δ*, dotted lines) defined by the time scale of the experiment as a function of the film boundary for (a) a smooth film and for (b) a rough film. At the fastest scan rates, the corresponding diffusion layer is confined within the film boundary (*δ*_1_). At intermediate scan rates, the diffusion layer passes through the roughness features of the film (*δ*_2_). At the slowest scan rates, the diffusion layer goes beyond the outermost film boundary (*δ*_3_). (c) Corresponding LSVs (*w*_*avg*_^1/2^ = 10, 2, 0.4) and (d) normalized peak current (*i*_*p*_) plot for a smooth film (solid line, shape factor = 100) and for a rough film (circles, shape factor = 2). The difference in current responses (Δ*i*_*p*_, shaded areas) at intermediate diffusion layer thicknesses allows for determination of the underlying film thickness distribution. Panels (a–d) adapted with permission from ref 43 under CC BY 3.0.

They parameterized the film thickness distribution with the 1-parameter Weibull distribution, where the “shape factor” defines its relative standard deviation. Numerical methods were used to validate edge effects resulting from hemispherical diffusion affecting the planar diffusion profiles as a function of the film section position (found to be negligible). They also demonstrated the method’s practical application by extracting the film thickness heterogeneity from smooth and heterogeneous redox-active films and compared their electrochemically-derived results with atomic force microscopy.

This work in combination with Aoki’s and other reports on modeling diffusional voltammetry at finite planar electrodes^44^ serves as a benchmark framework for developing an electroanalytical tool to extract structural information from electrodes with finite diffusion spaces: 1) dimensionless groups were developed, including heterogeneities, to describe the electrochemical system, 2) geometric simplifications of the electrochemical model were validated, 3) access to current simulations were provided, and 4) the method’s practical application was demonstrated with experimental studies.

## Nonplanar Finite Spaces

A review^45^ on voltammetry at non-planar electrodes, with a focus on rough and porous electrodes, included insights on mass transport mechanisms. It described the coexistence of semi-infinite diffusion from the bulk solution to the electrode surface and thin-layer diffusion within the porous matrix. Tichter and co-workers^46^ further examined the impact of mass transport on the voltammetric response at pillar-type electrodes, covering the transition from finite to semi-infinite diffusion. However, neither of these reviews explore the theory of diffusion at non-planar porous electrodes in-depth, nor do they address its application for pore structure characterization. The subsequent sections of this review aim to address this gap.

### Nonplanar Electrode Geometry Simplification

Non-planar electrodes, due to their complex geometry, require simulations in 3 dimensions to exactly represent the system. This is often negated for computational accessibility by simplifying the electrode geometries and/or diffusion domains to reduce the dimensionality of the problem.

### The Cylindrical Diffusion Domain Approximation

The cylindrical diffusion domain approximation is applicable to electrodes with a circular symmetric axis, which includes all the idealized geometric solids previously described. By simplifying the geometry of the volumetric diffusion space that surrounds the electrode into a cylinder, the problem is reduced from 3D Cartesian to 2D cylindrical with symmetry in the azimuthal coordinate. This simplification also means that, as every unit cell is identical (and with the assumption that minimal flux occurs between unit cells), only one electrode needs to be modeled and scaled to represent a complete array.

Although not strictly porous, an early example of the approximation’s use was by Amatore and co-workers^47^ in the context of uniform microdisk electrode arrays embedded within an insulating substrate (Figure 9a-b). By using a cylindrical diffusion volume (Figure 9c), they solved the problem analytically in cylindrical coordinates, which was the only feasible way for solving such a system at the time.

**Figure 9:**
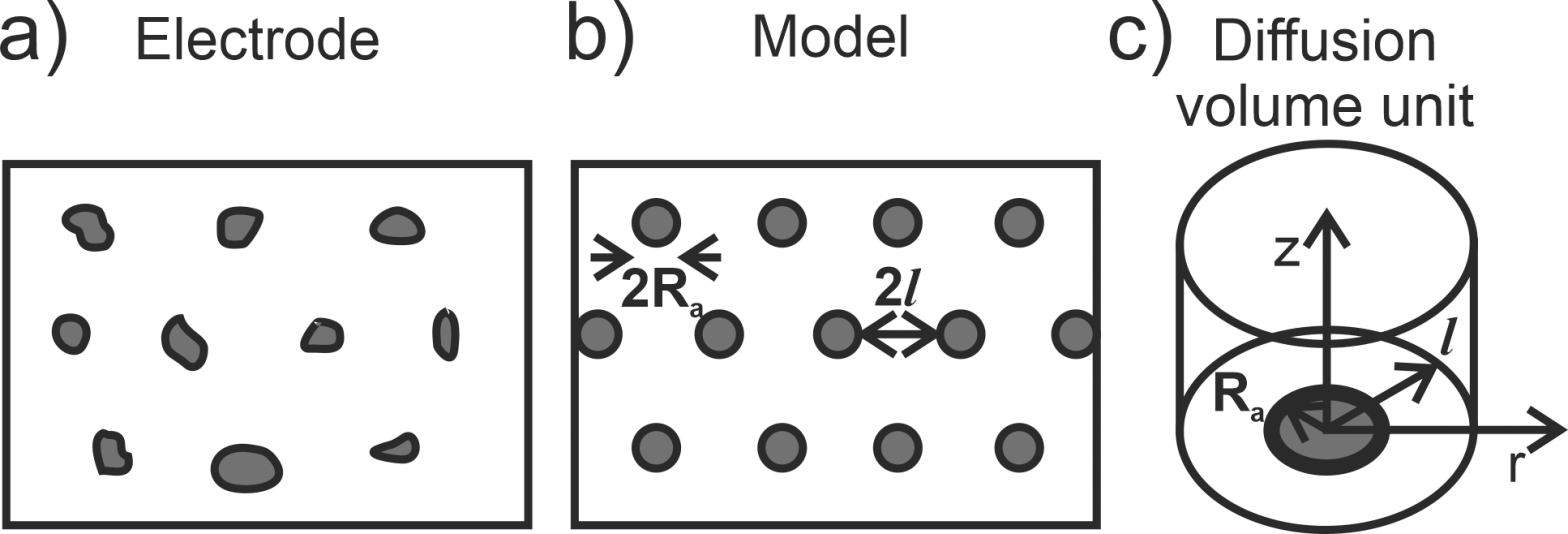
(a) The site arrangement for a microdisk array, (b) its idealized model representation, and (c) the simplified diffusion volume as a cylinder. Active sites are shown in gray, with the blocking film in white. *R*_*a*_ represents the average radius of the active site, while l is the average midpoint distance between two sites. Panels (a–c) adapted with permission from ref 47. Copyright 1983 Elsevier.

### Voronoi Tessellations as a Framework for Distributed Diffusion Domains

Distributed diffusion domains are also modeled using cylindrical diffusion domains under the theoretical framework of a Voronoi tessellation. An early example was by Davies and co-workers,^48^ who used this framework to model an electrode surface covered with non-uniform, inert blocking disks, however it has also been used in the context of porous electrodes, including distributed inverse opal,^35^ hollow tube,^35^ pillar array^35^ and particle array^49^ electrodes.

Voronoi cells are independent regions formed by the equidistant partitioning of a collection of points that lie within a plane (Figure 10a). Cylindrical cell equivalents are used to represent each of the Voronoi cells by equating the basal surface area of the space within which the point is placed (Figure 10b). These cylindrical cells are then modeled independently from one another by assuming zero flux between cells (Figure 10c).

**Figure 10:**
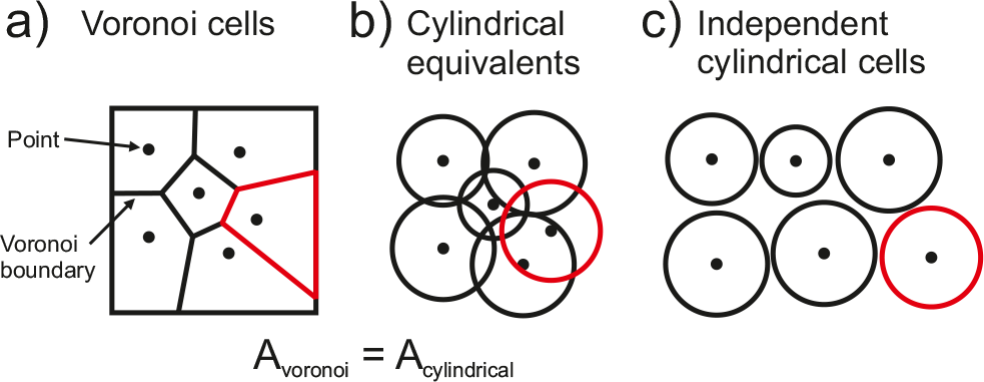
Schematic illustrating the process of generating independent cylindrical diffusion domains for points randomly positioned on a 2D plane, where points are divided into (a) Voronoi cells, (b) cylindrical cells, and (c) independent cylindrical cells.

A recent study by Oleinick and co-workers^50^ used the cylindrical approximation when exploring the theory of chronoamperometry at randomly distributed microdisk arrays. By performing a Voronoi tessellation, they demonstrated that using the cylindrical diffusion domain approximation results in a maximum error of only 5% when representing any randomly distributed microdisk array electrode.^51^ The primary source of this error stems from the assumption that each microdisk is centrally located within its corresponding Voronoi cell. In reality, the microdisks are often offset, making them less efficient at consuming redox-active species compared to the cylindrical cell equivalent, which leads to a reduced current output. These findings support the validity of also using the cylindrical approximation for modeling porous structures.

### Concave (−−) Inverse Opal Electrodes

Modeling an inverse opal electrode can be challenging, as the complex geometry at the interface between the inverse opal matrix and the bulk solution (Figure 11) prevents straightforward simplification based on a circular symmetry. Barnes and co-workers^52^ addressed this by simulating chronoamperometric and voltammetric currents for uniform inverse opal electrode matrices deposited on a flat conductive substrate, where electron transfer kinetics follow the Butler-Volmer rate expression.

**Figure 11:**
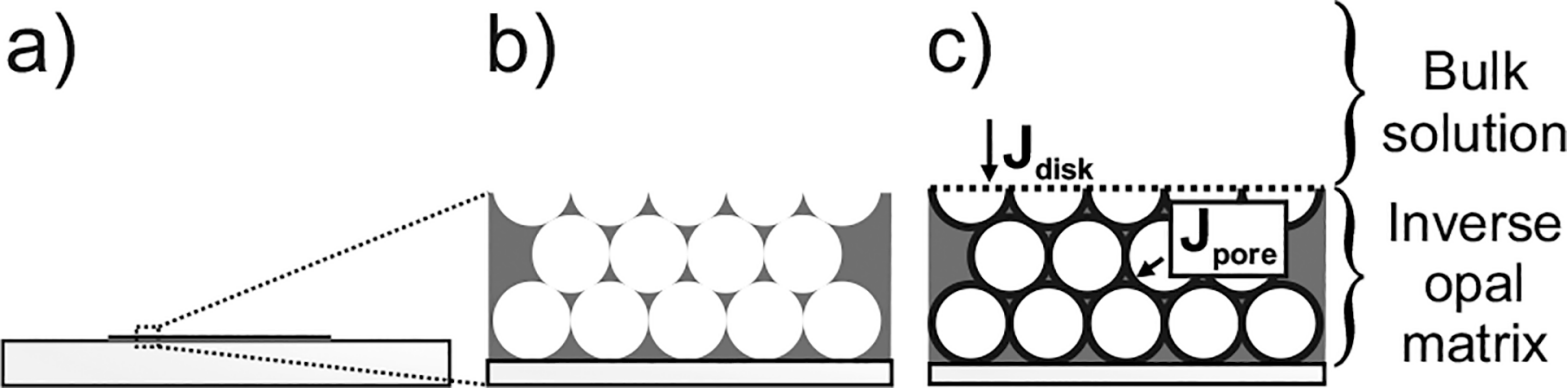
Schematic cross-section of a porous electrode composed of hollow spheres (inverse opals) on a flat conductive substrate. (a) Overview illustrating the significantly larger projected surface area relative to the matrix thickness, enabling the use of the “thin matrix” approximation. (b) Zoomed-in view showing the interconnected structure of the pores and interpore channels. (c) Representation of the electrochemical model, distinguishing independent pore and disk domains with their respective fluxes (*J*_*pore*_ and *J*_*disk*_).

They made two key assumptions: 1) the inverse opal matrix has a very large projected surface area relative to its thickness (Figure 11a), often referred to as the “thin matrix” approximation, which simplifies the model by ignoring edge effects; and 2) has pores within the inverse opal matrix that are interconnected by negligibly small channels, being large enough to allow solution access throughout the entire depth of the porous matrix (Figure 11b) but small enough that each pore can be approximated as a complete sphere (Figure 11c).

Their model separates the total current (*i*) into two independent contributions: 1) the pore currents (*i*_*pores*_) arising from the independent spherical “pores” of the inverse opal matrix, and 2) the disk current (*i*_*disk*_) originating from the projected plane of the inverse opal matrix. The total current response (*i*) is computed as the sum of these independent contributions (eq 17), and the total peak current (*i*_*p*_) is the maximum of this composite current (eq 18).

$$i=i_{pores}+i_{disk}$$ 17

$$i_{p}=\max\left(i_{pores}+i_{disk}\right)$$ 18

A correlation plot of the dimensionless peak current density (*j*_*p*_) versus the dimensionless scan rate (*w*) for a single pore (Figure 12a) enables the extraction of the homogeneous pore size ( l ) of an inverse opal electrode, assuming the relative contribution of the semi-infinite diffusion is negligible. Notably, Figure 12a also reveals the shift in diffusion regime within a pore from finite to semi-infinite as a function of *w*. This transition is marked by a change in the slope from 1.0 to 0.5 (Figure 12b). Finite diffusion dominates when *w* ^1/2^ < 0.56, semi-infinite diffusion is established when *w* ^1/2^ > 316, and a transitionary regime exists between these values. These values differ significantly from those reported by Aoki for the planar pore geometry, highlighting the faster diffusion dynamics within an inverse opal structure, where a much larger *w* ^1/2^ is required to achieve semi-infinite bulk diffusion.

**Figure 12:**
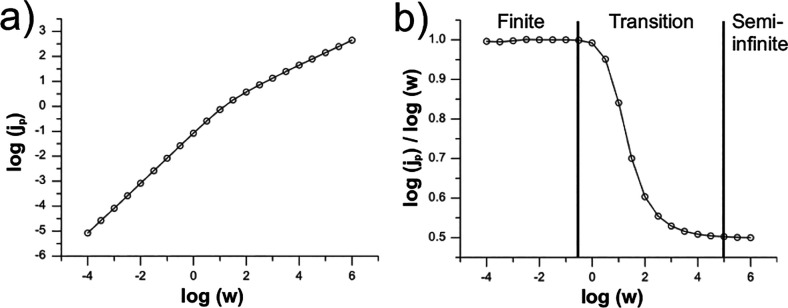
(a) Log of dimensionless peak current density (*j*_*p*_) simulated inside a sphere as a function of log of dimensionless scan rate (*w*). Ψ is set to 10^5^ to ensure electrochemical reversibility over the whole range. (b) Shows the slope of (a) with rough bounds for finite, transitionary, and semi-infinite diffusion. Panels (a, b) adapted with permission from ref 52. Copyright 2014 Elsevier.

Another study also describes modeling diffusional voltammetry at inverse opal electrodes,^53^ however not to the same degree of detail as that of Barnes and co-workers^52^ as it neglected the semi-infinite disk contribution.

### Concave (−) Hollow Cylinder Electrodes

Hollow cylinders are typically modeled using the cylindrical diffusion domain approximation described earlier. Menshykau and co-workers^54^ developed the theoretical framework for voltammetry at uniform hollow cylinders, where electron transfer is governed by the Butler-Volmer rate expression. They investigate how geometric parameters such as the pore depth (*L*_*cyl*_ = *l*_*cyl*_/ l ) and pore size (*R*_*max*_ = *r*_*max*_/ l ) influence the voltammetric response (the physical model is shown in Figure 13a).

**Figure 13:**
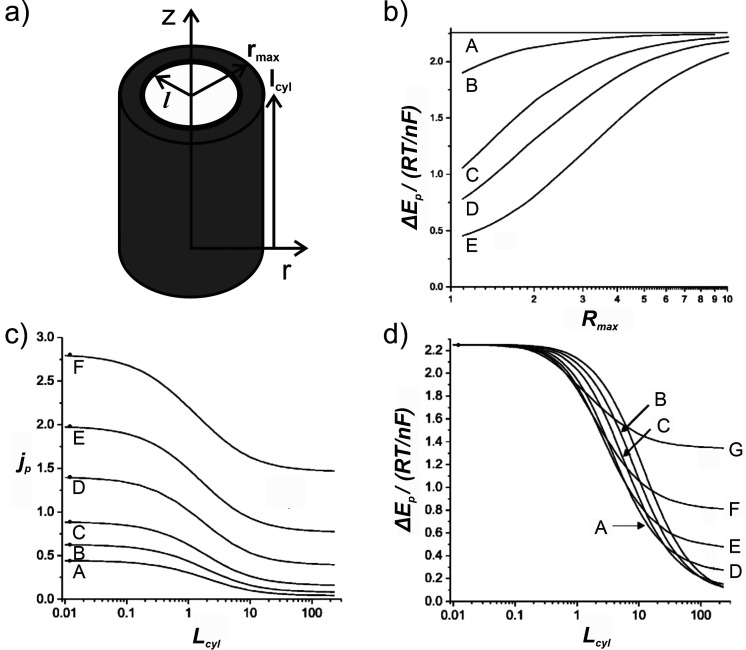
(a) Cylindrical diffusion domain equivalent of a hexagonally-arranged hollow cylinder array in cylindrical coordinates. (b) Reversible peak-to-peak separation versus pore size *R*_*max*_ at (A) a flat macroelectrode and (B–E) different cylinder heights *L*_*cyl*_ = 1, 2, 4, and 10. (c) Reversible peak current density and (d) peak-to-peak separation versus cylinder depth *L*_*cyl*_ at different scan rates (A–G), *w* = 0.1, 0.2, 0.4, 1, 2, 4, and 10, with dots showing flat macroelectrode values. Panels (b–d) adapted with permission from ref 54. Copyright 2008 John Wiley & Sons.

They provided detailed correlation plots of peak current density (*j*_*p*_) and peak-to-peak separation (Δ*E*_*p*_) as functions of dimensionless scan rate *w*, pore depth *L*_*cyl*_ and pore size *R*_*max*_ for both reversible (Figure 13b-d) and non-reversible electrode kinetics. Notably, an increase of the pore depth beyond *L*_*cyl*_ > 0.1 causes a decrease in *j*_*p*_ (Figure 13c) and Δ*E*_*p*_ (Figure 13d) as the response transitions from semi-infinite to finite diffusion. By correlating experimental data with their simulated results, it is possible to estimate the pore depth and pore size, assuming these parameters are homogeneous across the array.

Other studies also describe modeling diffusional voltammetry at hollow cylinder electrodes,^55^ including investigations into how diffusion behavior varies with scan rate.^56^ However, they did not specifically examine the influence of pore structure on the current response, as was done by Menshykau and co-workers.^54^

### Convex (+) Pillar Array Electrodes

Pillar arrays are also typically modeled using the cylindrical diffusion domain approximation, which assumes that the array’s projected surface area is sufficiently large relative to the pillars’ height so that semi-infinite diffusion to the sides of the array is negligible (Figure 14a). Dickinson and co-workers,^57^ Henstridge and co-workers,^58^ and Prehn and co-workers,^59^ described the theoretical framework for simulating chronoamperometric and/or voltammetric currents at uniform pillar arrays deposited on a flat conductive substrate, using a nearly identical physical model to that shown in Figure 14b.

**Figure 14:**
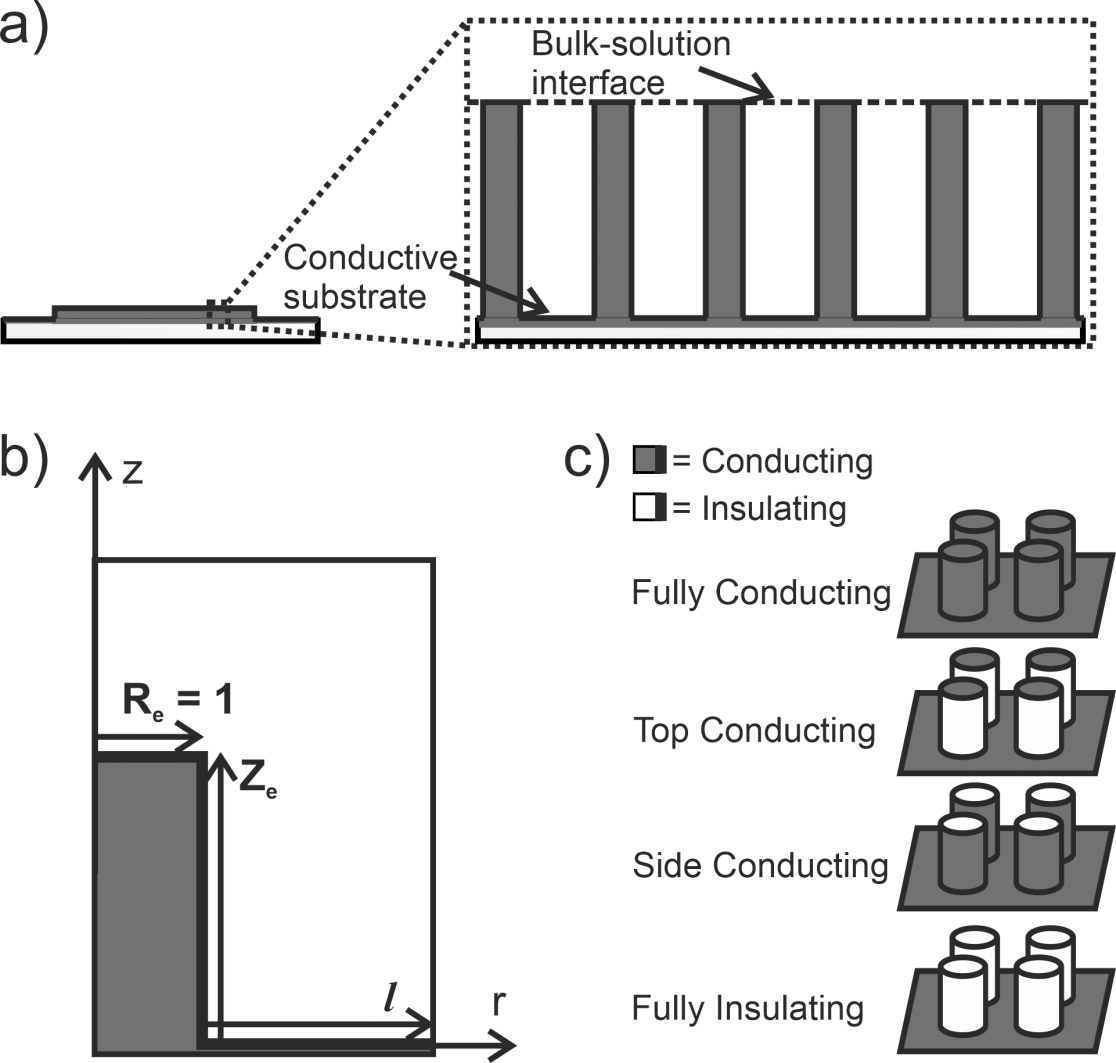
(a) Schematic cross-section of a pillar array electrode on a conductive, flat substrate and zoomed-in view highlighting the edge effects at the conductive substrate and bulk-solution interface. The larger projected surface area relative to its thickness allows the “thin matrix” approximation, neglecting side diffusion. (b) Schematic of the simulation space for a fully conducting, uniform pillar array. (c) Schematic illustrating the various conductivity cases for a pillar array. Panel (b) adapted with permission from ref 58. Copyright 2012 Springer Nature BV. Panel (c) adapted from ref 57. Copyright 2008 American Chemical Society.

Dickinson and co-workers^57^ investigated the influence of edge effects by simulating various conductivity scenarios for pillar arrays with reversible kinetics, considering fully conducting pillars, as well as pillars conducting only at the top, side, or base (Figure 14c). They examined how dimensionless geometric parameters affect the current response, such as the dimensionless pillar height (*Z*_*e*_) (eq 19) where *z*_*e*_ is the physical pillar height and *r*_*e*_ is the pillar radius, and the surface coverage (*θ*) (eq 20), where *R*_*e*_ is the dimensionless pillar radius and *R*_*max*_ is the dimensionless radius of the cylindrical domain, with *R*_*max*_ = *R*_*e*_ + l . Since *R*_*e*_ = 1, *R*_*max*_ directly corresponds to the interpillar distance l .

$$Z_{e}=z_{e}/r_{e}$$ 19

$$\theta =\pi {R_{e}}^{2}/\pi {R_{\max}}^{2}$$ 20

Their simulations revealed that for fully conducting pillars with a dimensionless height of *Z*_*e*_ = 1, the current response remained largely unaffected for interpillar distances greater than *R*_*max*_ > 10. However, at *Z*_*e*_ = 10 (and presumably for even taller pillars), the current response became completely dependent on *R*_*max*_, suggesting a potential method for determining the interpillar distance in uniform pillar arrays of this height or greater. Additionally, at *Z*_*e*_ = 10, the current response for fully conducting and side conducting pillar arrays overlapped, indicating that the specific geometry of the pillar top has negligible influence when correlating experimental data with simulations (see Figure 16a–c as an example).

Henstridge and co-workers^58^ further investigated the influence of interpillar distance (*R*_*max*_) and pillar height (*Z*_*e*_) on the voltammetric peak current (*i*_*p*_) for fully conducting pillar arrays, with the electron transfer kinetics described by the Butler-Volmer rate expression.

Linear sweep voltammograms were simulated at a single, low scan rate (*w* = 0.01) representing the finite diffusion regime, for various values of *R*_*max*_ and *Z*_*e*_. Correlation plots revealed a strong dependence of both the peak current *i*_*p*_ (Figure 15a) and peak potential (*E*_*p*_) (Figure 15b) on these geometric parameters. However, due to the parabolic nature of the curves, multiple values of *R*_*max*_ could result in identical values of *i*_*p*_ and *E*_*p*_ for a given value of *Z*_*e*_ and *w*. This ambiguity means that neither plot alone is sufficient to uniquely determine *R*_*max*_ or *Z*_*e*_. Nevertheless, by analyzing both plots together, it may be possible to resolve either *R*_*max*_ or *Z*_*e*_ if the other is known.

**Figure 15:**
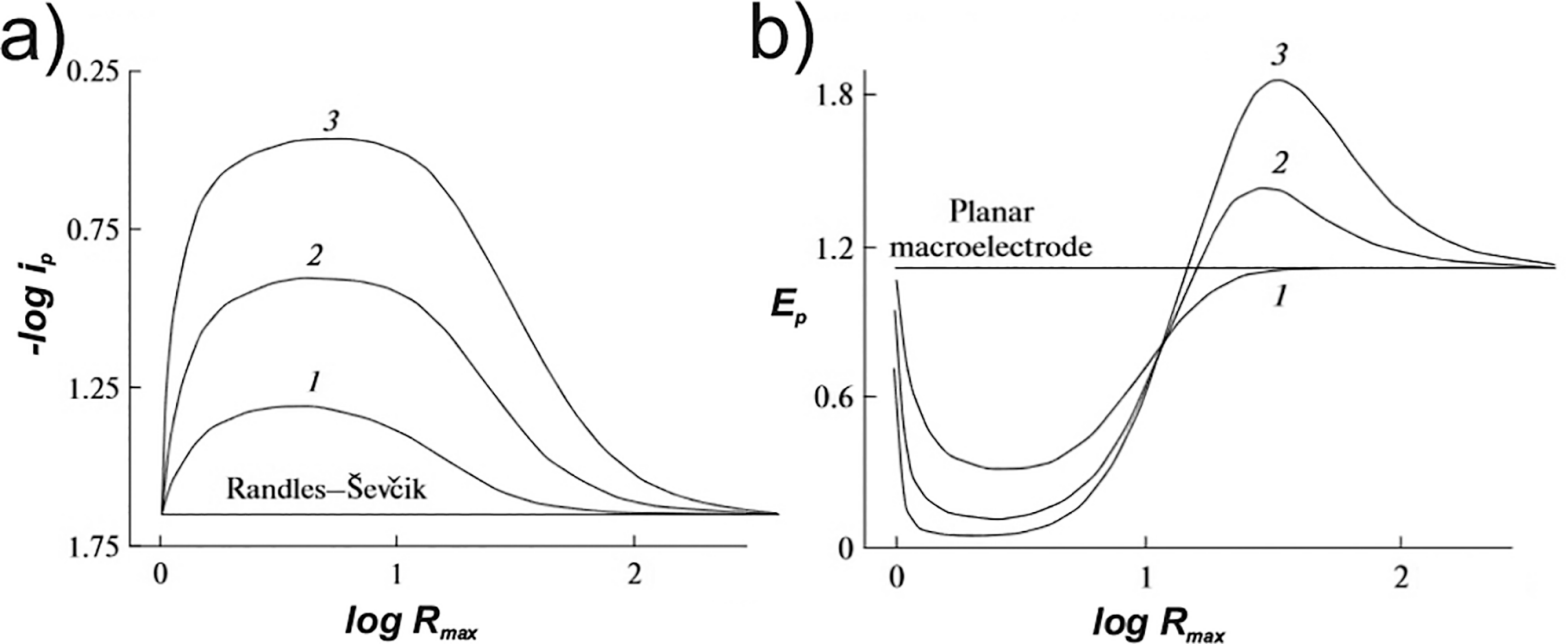
Scaled (a) peak current and (b) peak potential as a function of interpillar distance *R*_*max*_ for a reversible redox couple at various pillar heights (*Z*_*e*_ = 30 (1), 100 (2), and 300 (3)) simulated at a scan rate of *w* = 0.01. *R*_*max*_ is defined as the sum of the electrode radius and diffusion domain length $\left(R_{e}+l\right)$ . Panels (a–b) adapted with permission from ref 58. Copyright 2012 Springer Nature BV.

Prehn and co-workers^59^ simulated cyclic voltammograms at pillar array electrodes, where the electron transfer kinetics follows the Butler-Volmer model, to electrochemically determine the interpillar distances of electrodes experimentally. They fabricated pillar array electrodes with three different interpillar distances (50, 100, and 200 μm) and varying heights (5–15 μm) confirmed through microscopy and profilometry (Figure 16a-c). By recording peak current densities across multiple scan rates and plotting peak current density (*J*_*p*_) versus the square root of the scan rate (*v* ^1/2^) (Figure 16d-f), they demonstrated a practical method for extracting the interpillar distance l of a uniform pillar array by fitting the experimental data to simulated currents. Figure 16d-f also highlights that as the interpillar distance *R*_*max*_ increases, the peak currents approach those of a macroelectrode, represented as the Randles-Ševčík relationship.

**Figure 16:**
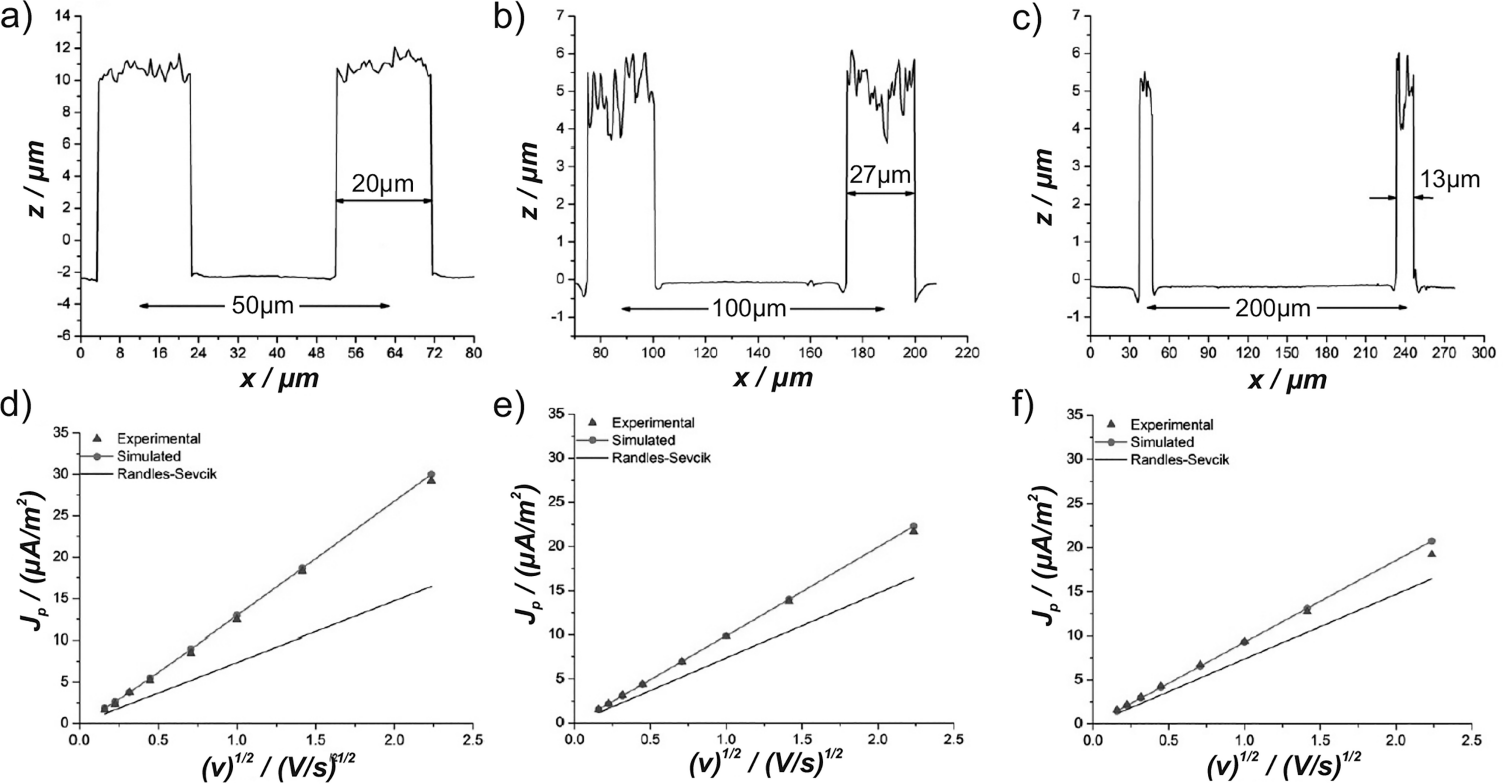
(a–c) Confocal microscopy images of three representative micropillar array electrodes illustrating the interpillar distance, pillar height, and pillar tip roughness. (d–f) Experimental (blue triangles) and simulated (red dots) *J*_*p*_ vs *v* ^1/2^ for microcylinder array electrode radius and height of 10 μm and interpillar distances of (d) 50 μm, (e) 100 μm, and (f) 200 μm. The response of an equivalent flat electrode is shown for comparison (black line). Panels (a–f) adapted with permission from ref 55. Copyright 2013 Elsevier.

Smith and co-workers^32^ correlated experimental voltammograms with simulations at graphite felt electrodes using a novel data processing approach that simultaneously determined the average pore size ( l ) and electrochemical surface area (*S*). They approximated the diffusion space around multiple fibers as a cylindrical pore, which they further simplified to a thin-layer plane electrode model (Figure 17a). While this simplification deviates from a true pillar geometry and is thus likely to introduce significant error, it enabled the use of the commercially available software DigiElch for performing simulations. They validated their method experimentally by recording multiple cyclic voltammograms at a graphite felt electrode (one example is shown in Figure 17b) and generating plots of equivalent surface area versus average pore size at different scan rates (Figure 17c). From the crossover point highlighted by the red arrow, they extracted parameter values of approximately *S* = 50 cm^2^ and l = 30 μm (Figure 17c).

**Figure 17:**
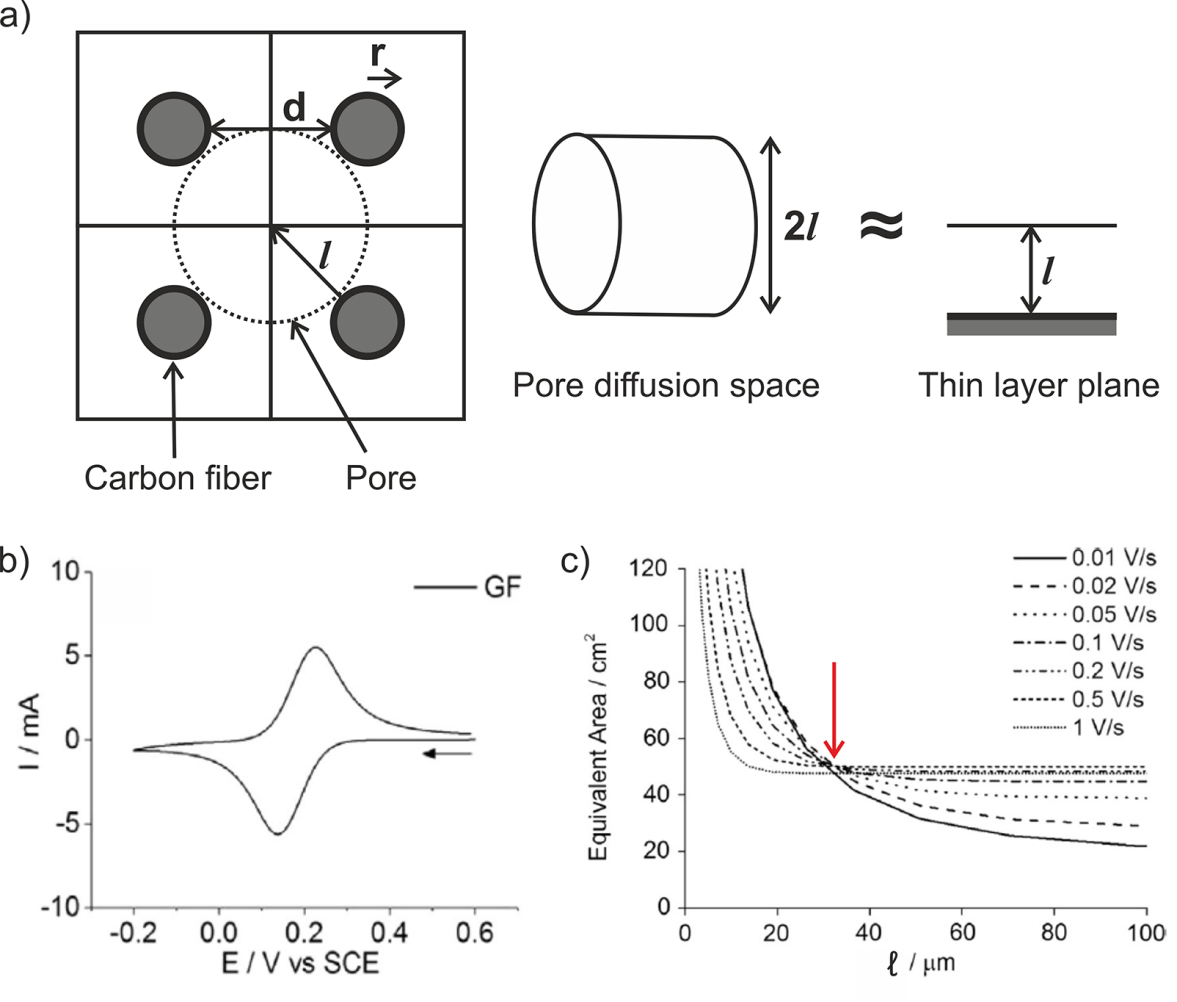
(a) Schematic representation of the unit cells for evaluating pore radius ( l ) and fiber-to-fiber distance (*d*), illustrating the approximation of multiple fibers as a pore treated as a thin layer plane electrode. (b) Cyclic voltammograms of a graphite felt electrode recorded in 0.1 mM ferricyanide (0.1 M KNO_3_) at a scan rate of 50 mV/s. (c) Simulated plots of equivalent surface area versus average pore radius ( l ) at various scan rates, based on the cathodic peak currents from the graphite felt electrode under the same conditions. The red arrow indicates the crossover point for extracting the surface area and pore size. Panels (b, c) adapted with permission from ref 32. Copyright 2015 Elsevier.

Other studies also describe modeling diffusional voltammetry at pillar array electrodes, including investigations on how the peak current varies with multiple CV cycles^60^ and how fitting cyclic voltammograms can be used for simultaneous investigations of the kinetics and pore structure of carbon felt electrodes.^61^

### Convex (++) Particle Array Electrodes

Particle arrays are typically modeled using a similar approach to pillar arrays, applying the cylindrical diffusion domain approximation to represent a section of each particle, while assuming that semi-infinite diffusion at the edges of the array is negligible. Notable contributions by Belding and co-workers^49^ and Streeter and co-workers^62^ developed the theorical framework for chronoamperometry and voltammetry at randomly distributed particle arrays employing a Voronoi tessellation to define individual diffusion domains.

Belding and co-workers^49^ modeled the chronoamperometric and voltammetric currents (physical model shown in Figure 18a) by weighing them with a randomly distributed domain size (*R*_*d*_ = *r*_*e*_ + l ) (Figure 18b). Their simulations demonstrated that the shape of the chronoamperogram evolves with changes in the mean domain radius ⟨*R*_*d*_⟩ of the array (Figure 18c), providing a method to estimate ⟨*R*_*d*_⟩, assuming the particle array can be adequately represented by their random distribution. However, it is arguable that their random distribution function as shown in Figure 18b is relatively homogeneous. A similar analysis can be applied to the peak current of both reversible (Figure 18d) and non-reversible voltamograms. However, their study did not explore how variations in domain size distribution might influence the overall current response.

**Figure 18:**
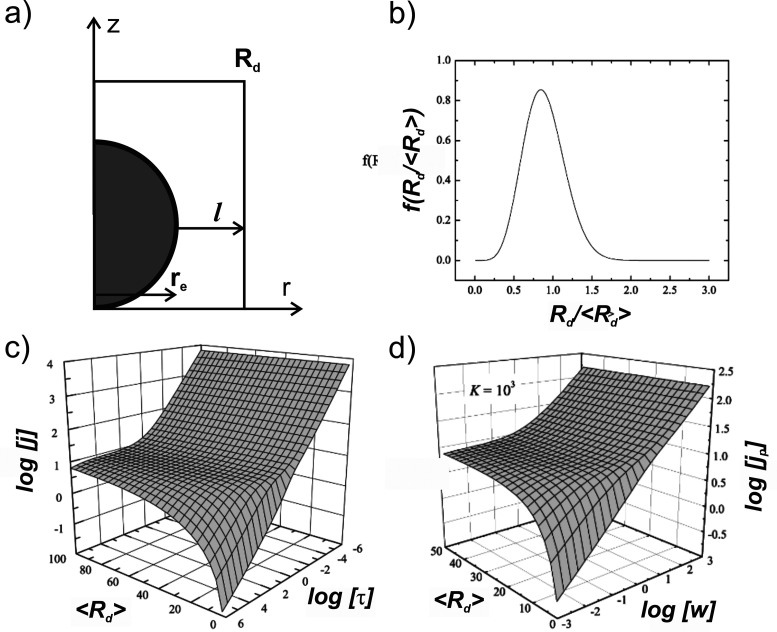
(a) Schematic of the simulation space for a spherical particle defined in cylindrical coordinates. (b) Size distribution for a random array. (c) Chronoamperometry current *J* as a function of mean domain radius ⟨*R*_*d*_⟩ and time *τ*. (d) Reversible CV peak current *J*_*peak*_ as a function of mean domain radius ⟨*R*_*d*_⟩ and scan rate *w*. Panels (b–d) adapted from ref 49. Copyright 2010 American Chemical Society.

Streeter and co-workers^62^ investigated reversible chronoamperometry and voltammetry at a randomly-distributed array of spherical particles, where the averaged current is weighted by varying diffusion domain sizes. They showed that the relationship between peak current and the square root of scan rate deviates from the classical planar electrode behavior as a function of interparticle distance ( l ), although their analysis appears to assume a uniform particle distribution. Using diffusional voltammetry, they characterized a palladium particle array, assuming homogeneous particle size, by recording peak currents across multiple scan rates to extract l , showcasing a practical application of using diffusional voltammetry to characterize particle arrays.

Other studies also describe modeling diffusional voltammetry at particle array electrodes, including investigations that highlight how distributions of particles can display an apparent catalytic effect due to changing diffusion regimes^63^ as well as the change in current response as spheroid/hemispheroid particles become oblate or prolate in curvature.^64^

## A General Framework for Multiple Nonplanar Geometries

Until this point, the studies discussed have focused exclusively on individual non-planar geometries, without addressing how current responses vary between different porous electrode geometries. Tichter and co-workers^35^ produced a study that attempts such a comparison, simulating cyclic voltammograms for a range of electrode geometries, including internal spheres (inverse opals), internal cylinders (hollow cylinders), planes (films), and external cylinders (pillar arrays), each incorporating a statistical distribution of pore sizes. Simulations are made available through an open access app.^35^

The aim of their study was to extract both heterogeneous electron transfer kinetics and homogeneous chemical kinetics by fitting the full experimental CVs of a porous electrode to a simulated CV at a low scan rate (ensuring finite diffusion conditions). A key finding was that both electrode kinetics and the pore size distribution similarly influence the CV shape, meaning that knowledge of one parameter is necessary to determine the other.

Figure 19 shows how, at a constant electrochemical rate, the CV profile for the different porous electrodes across the four geometries vary as a function of the pore size distribution. Interestingly, for planar films and pillar arrays, increasing heterogeneities led to a decreased current response (Figure 19a-b), while the opposite trend was observed for inverse opals and hollow cylinders (Figure 19c-d). The decrease in peak current for heterogeneous planar electrodes align with the finding reported by Buesen and co-workers.^43^ Overall, their results demonstrate that if electrode kinetics are known and constant throughout an experiment, it is possible to extract the pore size distribution across these four electrode geometries.

**Figure 19:**
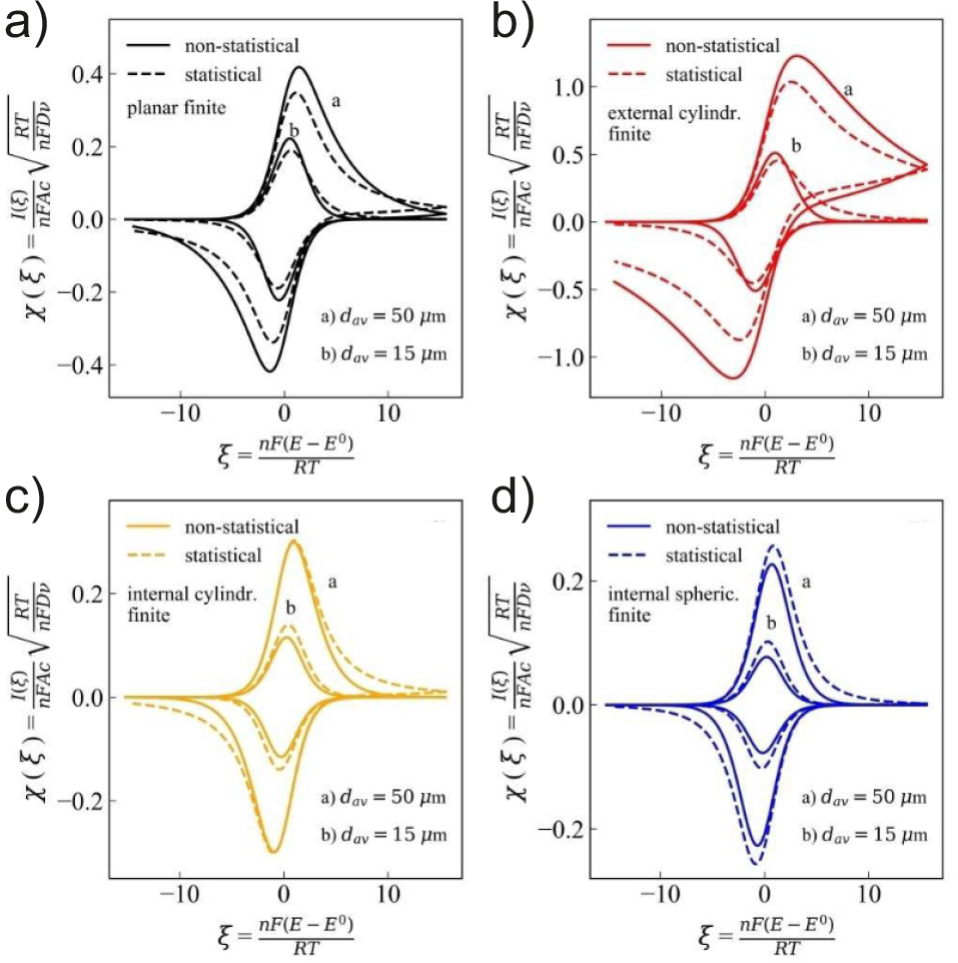
Simulated dimensionless current responses versus dimensionless potentials for porous electrodes possessing statistically distributed diffusion domains with (a) planar finite (black), (b) cylindrical external finite (red), (c) cylindrical internal finite (orange), and (d) spherical internal finite (blue) symmetry. “Non-statistical” refers to homogeneously distributed domains, while “statistical” refers to randomly distributed domains. Panels (a–d) adapted with permission from ref 35 under CC BY 4.0.

They attempted to discern the change in electrode kinetics post-application for a folded platinum mesh (Figure 20a) and a technically-relevant carbon felt electrode (Figure 20b) by fitting experimental data to simulated currents using their pillar array model with a randomly distributed diffusion domain. The radius of the fibers was determined by inspecting a scanning electron microscope image. However, as their model only included the bulk pore (without edge effect contributions occurring externally from the bulk solution), the low surface area platinum mesh led to a (self-described) poor fit between simulated and experimental voltammograms, however it is arguably still highly reasonable. In contrast, the high surface area of the carbon felt mitigated the impact of edge effects on the current response, leading to an excellent fit between simulated and experimental voltammograms.

**Figure 20:**
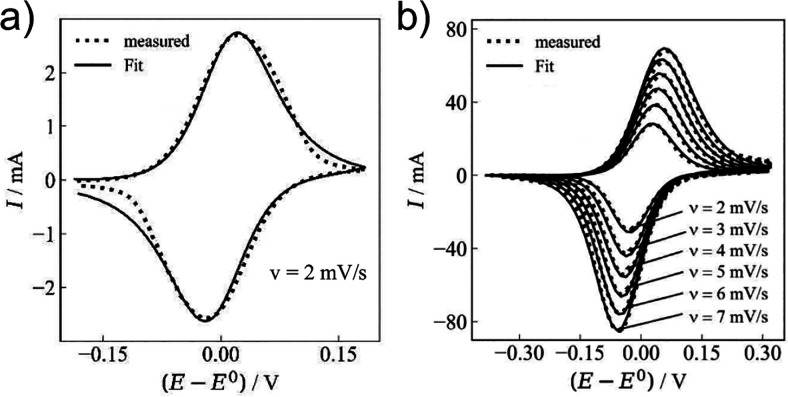
Fit (solid curves) and measured (dots) data for (a) a random Pt-wire network and (b) GFD-carbon felt electrode described by a statistical external cylindrical diffusion domain. Panels (a, b) adapted with permission from ref 35 under CC BY 4.0.

By ignoring edge effects, they were able to derive analytical solutions for the electrochemical problem across various complex electrode geometries. However, while they provide models for four different pore geometries, the shape of a finite CV alone does not offer any clear indication of which model to apply. As a result, determining the pore geometry in advance, likely through a non-electrochemical technique, is necessary to choose the appropriate model for the fitting procedure.

Other studies also describe modeling diffusional voltammetry at porous electrodes,^65,66^ however they do not include heterogeneities or experimental validations, nor do they directly compare current responses between the different pore structures as was done by Tichter and co-workers.^35^

## Conclusions/Outlook

Significant progress has been made in modeling various electrode geometries with finite diffusion spaces, with key contributions from Aoki, Buesen, Plumeré, Amatore, Oleinick, Compton and Tichter. Collectively, these studies have advanced our understanding of diffusional voltammetry in porous electrodes exhibiting finite diffusion by relating simulated current responses to pore structure in the form of correlation plots. Furthermore, these studies provide a basis for experimental pore structure characterization by fitting experimental data to simulated data, which is provided either via access to simulations with an app or the correlation plots themselves.

The work on planar porous electrodes by Aoki and Buesen serves as a benchmark electroanalytical framework for using the current response to characterize porous electrodes. They incorporated structural heterogeneities in the model, formed dimensionless groups, validated geometric model simplifications, provided access to simulate currents, and demonstrated practical application by including experimental studies.

The closest match for non-planar porous electrodes was provided by Tichter, however further advancements are needed for these geometries by comprehensively investigating edge effects and characterizing electrodes experimentally. The collection of studies highlighted in this review on non-planar geometries together provide a strong foundation for establishing an electroanalytical framework comparable to that available for planar electrodes.

Finally, further efforts and consideration should be given to generalize pore structure parameterization if one is to develop a universal electrochemical tool capable of characterizing all the geometries described in this review with a single model, or further yet, highly-irregular, highly-complex porous electrodes that are beyond what can be described with idealized geometric solids. As discussed in this review, one promising approach involves utilizing the curvature as a unifying descriptor.

## Supplementary Material



## Notes

Mathematical derivations, calculations, and general solution for geometry-dependent characteristic diffusion times (PDF)
