# Supplementary material for: Diffusional Voltammetry in Finite Spaces
Source: ACS Electrochem. 2025 Jun 2;1(8):1258–73. doi: 10.1021/acselectrochem.5c00091 (PMC12337096; doi:10.1021/acselectrochem.5c00091)
Supplement: Supplementary file 1 [file ec5c00091_si_001.pdf]

# Supporting Information

## Diffusional Voltammetry in Finite Spaces

Yoshua H. Moore, Ben A. Johnson, Nicolas Plumeré*

Technical University of Munich (TUM), Campus Straubing for Biotechnology and Sustainability, Uferstraße 53, 94315 Straubing, Germany

*Email: nicolas.plumere@tum.de

## Contents

**Relating Diffusion Dynamics to Pore Structure** **S2**

**1 Problem set-up** **S2**

**2 Axisymmetric diffusion in one dimension** **S2**

**3 Eigenfunction expansion solution** **S3**

**4 Current response and Nernst diffusion layer thickness** **S3**

**5 Eigenfunctions and characteristic equations for eigenvalues** **S4**

**6 General solution for characteristic diffusion time** **S4**

6.1 Convex diffusion . . . S5

6.2 Concave diffusion . . . S5

**7 Diffusion time in different geometries** **S5**

7.1 Plane . . . S5

7.2 Cylinder . . . S6

7.2.1 Concave cylinder . . . S6

7.2.2 Convex cylinder . . . S6

7.3 Sphere . . . S7

7.3.1 Concave sphere . . . S8

7.3.2 Convex sphere . . . S8

**8 Summary** **S9**

# Relating Diffusion Dynamics to Pore Structure

## 1 Problem set-up

$$\mathrm{P} + e^- \rightleftharpoons \mathrm{Q} \tag{S1}$$

Consider an electrode surface at $x = a$ ($0 < a < \infty$) with either planar, cylindrical, or spherical geometry and a surface area $S$. A freely-diffusing oxidized species P is initially present at a concentration $C^0$. A large potential step is applied, initiating the reduction of P, such that its surface concentration at the electrode is instantaneously stepped to zero: $C(a,t) = 0$ for $t > 0$. Let the diffusion coefficient of P be given by $D$. There is a closed outer boundary at $x = b$ ($0 \leq b < \infty$, $b \neq a$), where $\partial_x C(b,t) = 0$. The size of the finite domain is $\ell = |a - b|$. **For concave diffusion: $b < a$; and for convex diffusion: $b > a$.** Diffusion inside a pore and semi-infinite diffusion are represented by the limiting cases $b = 0$ and $b \to \infty$, respectively.

## 2 Axisymmetric diffusion in one dimension

For one-dimensional diffusion in axisymmetric curvilinear coordinates, the diffusion of P is governed by

$$\frac{\partial C}{\partial t} = D\mathcal{L}_{x,d}C, \tag{S2}$$

where the general Laplacian operator is

$$\mathcal{L}_{x,d} = \frac{1}{x^{d-1}}\frac{\partial}{\partial x}\left(x^{d-1}\frac{\partial}{\partial x}\right), \tag{S3}$$

with $d \in \{1,2,3\}$. The cases $d = 1$, $d = 2$, and $d = 3$ correspond to planar, cylindrical, and spherical coordinates, respectively. The initial condition is

$$C(x,0) = C^0. \tag{S4}$$

We also have homogeneous Robin boundary conditions given by

$$\alpha_i C(i,t) + \beta_i \frac{\partial C}{\partial x}(i,t) = 0. \tag{S5}$$

For the boundary condition at $x = a$, the coefficients are $\alpha_a = 1$, $\beta_a = 0$, and for the boundary condition at $x = b$, the coefficients are $\alpha_b = 0$, $\beta_b = 1$. The current is defined as

$$i = FSD\frac{\partial C}{\partial x}(a,t), \tag{S6}$$

where $F$ is Faraday's constant. We define the following dimensionless variables:

$$\Theta = \frac{C}{C^0}, \quad \chi = \frac{x}{a}, \quad \eta = t\frac{D}{a^2}, \quad \gamma = \frac{b}{a}, \quad \psi = \frac{i}{FSD\frac{C^0}{a}}, \quad L = \frac{\ell}{a} = |1-\gamma|. \tag{S7}$$

The dimensionless problem to solve becomes

$$\frac{\partial \Theta}{\partial \eta} = \mathcal{L}_{\chi,d}\Theta, \tag{S8}$$

$$\Theta(\chi, 0) = 1, \tag{S9}$$

$$\Theta(1,\ \eta) = 0, \qquad \frac{\partial \Theta}{\partial \chi}(\gamma, \eta) = 0, \tag{S10}$$

$$\psi = \frac{\partial \Theta}{\partial \chi}(1, \eta). \tag{S11}$$

## 3 Eigenfunction expansion solution

$\mathcal{L}_{\chi,d}$ is a Sturm-Liouville operator of the form

$$\mathcal{L}_{\chi,d} = \frac{1}{w(\chi)}\frac{\partial}{\partial \chi}\left(p(\chi)\frac{\partial}{\partial \chi}\right) + q(\chi), \tag{S12}$$

where $w(\chi) = p(\chi) = \chi^{d-1}$, and $q(\chi) = 0$, which admits the inner product

$$\langle f, g\rangle = \pm \int_1^{\gamma} f(\chi)g(\chi)w(\chi)d\chi. \tag{S13}$$

$\langle f, g\rangle$ is defined as positive for $\gamma > 1$ and negative for $\gamma < 1$ (to switch the integration limits). We define an eigenfunction basis as the set of solutions to the eigenvalue problem

$$\mathcal{L}_{\chi,d}\phi_n(\chi) = -\lambda_n^2\phi_n(\chi), \tag{S14}$$

$$\phi_n(1) = 0, \quad \phi_n'(\gamma) = 0, \tag{S15}$$

where $\lambda_n$ are the eigenvalues and $\phi_n$ are the eigenfunctions. This operator is self-adjoint with homogeneous Robin boundary conditions. Therefore, the solution to the diffusion problem is sought in the form of an eigenfunction expansion given by

$$\Theta(\chi, \eta) = \sum_n A_n(\gamma)\exp\left(-\lambda_n^2\eta\right)\phi_n(\chi, \gamma), \tag{S16}$$

$$A_n(\gamma) = \frac{\langle \phi_n, 1\rangle}{\langle \phi_n, \phi_n\rangle}. \tag{S17}$$

The eigenfunctions for a given coordinate system are only a function of $\chi$ and $\gamma$, which we denote as $\phi_n(\chi, \gamma)$.

## 4 Current response and Nernst diffusion layer thickness

From Eq. S11, the dimensionless current is

$$\psi = \sum_n A_n(\gamma)\exp\left(-\lambda_n^2\eta\right)\phi_n'(1, \gamma). \tag{S18}$$

The dimensionless Nernst diffusion layer thickness is

$$\tilde{\delta} = \frac{\delta}{a}, \tag{S19}$$

which is defined by a linear approximation to the concentration profile about $\chi = 1$. This leads to

$$\tilde{\delta} = |\psi^{-1}|. \tag{S20}$$

## 5 Eigenfunctions and characteristic equations for eigenvalues

For a plane ($d = 1$):

$$\phi_n = \frac{\sin(\lambda_n(1-\chi))}{\sin(\lambda_n)}, \quad \cos(\lambda_n(\gamma - 1)) = 0. \tag{S21}$$

For a cylinder ($d = 2$):

If $\gamma = 0$,

$$\phi_n = J_0(\lambda_n \chi), \quad J_0(\lambda_n) = 0. \tag{S22}$$

If $\gamma > 0$,

$$\phi_n = \frac{Y_0(\lambda_n\chi)\,J_0(\lambda_n) - Y_0(\lambda_n)\,J_0(\lambda_n\chi)}{J_0(\lambda_n)}, \quad J_0(\lambda_n)\,Y_1(\lambda_n\gamma) - J_1(\lambda_n\gamma)\,Y_0(\lambda_n) = 0. \tag{S23}$$

$J_\nu$ and $Y_\nu$ are Bessel functions of the first and second kind of order $\nu$, respectively.

For a sphere ($d = 3$):

$$\phi_n = \frac{\sin(\lambda_n(\chi - 1))}{\lambda_n \chi}, \quad \tan(\lambda_n(\gamma - 1)) = \gamma\lambda_n. \tag{S24}$$

## 6 General solution for characteristic diffusion time

We define the characteristic diffusion time, $\tau$ as the time required for the diffusion layer to reach the finite boundary at $x = \gamma$, i.e., the time it takes to traverse the entire finite domain of length $\ell$. This is geometry dependent and allows for extracting geometric information from the current response. In dimensionless form this is given by

$$\tilde{\tau} = \tau\frac{D}{\ell^2}. \tag{S25}$$

The characteristic diffusion time will scale as the longest time scale in the expansion (Eq. S18), which is given by the smallest eigenvalue $\lambda_0$. Therefore, using Eq. S20, we find

$$L = |\psi(\tilde{\tau}, \lambda_0)^{-1}| = \left(|A_0(\gamma)\phi_0'(1,\gamma)| \exp\left(-\lambda_0^2\tilde{\tau}\right)\right)^{-1}, \tag{S26}$$

which can be solved explicitly for $\tilde{\tau}$ given that $L = |1-\gamma|$:

$$\tilde{\tau}(\gamma) = \frac{\ln|(1-\gamma)A_0(\gamma)\phi_0'(1,\gamma)|}{\lambda_0^2}. \tag{S27}$$

The eigenvalues $\lambda_n$, and therefore $\tilde{\tau}$, are uniquely determined by the set of parameters $(d, \gamma)$. This means that Eq. S27 depends only on the geometry, represented by $d$, and the parameter $\gamma$, which distinguishes between concave diffusion ($\gamma < 1$) and convex diffusion ($\gamma > 1$).

### 6.1 Convex diffusion

Let $\gamma = 2$. This corresponds to convex diffusion within a hollow shell of length $\ell$ (with the dimensionless finite domain size given by $L = 1$).

$$\tilde{\tau} = \frac{\ln|A_0(2)\phi_0'(1,2)|}{\lambda_0^2}. \tag{S28}$$

### 6.2 Concave diffusion

Let $\gamma = 0$. This describes concave diffusion inside a pore of size $\ell$ (with the dimensionless finite domain size given by $L = 1$).

$$\tilde{\tau} = \frac{\ln|A_0(0)\phi_0'(1,0)|}{\lambda_0^2}. \tag{S29}$$

## 7 Diffusion time in different geometries

### 7.1 Plane

We begin with the eigenvalue expression for $d = 1$:

$$\lambda_n = \frac{\pi\left(n+\frac{1}{2}\right)}{\gamma-1}, \quad n \in \mathbb{Z}. \tag{S30}$$

The corresponding eigenfunctions $\phi_n(\chi, \gamma)$ are given by

$$\phi_n(\chi,\gamma) = \csc\left(\frac{\pi\left(n+\frac{1}{2}\right)}{\gamma-1}\right)\sin\left(\frac{\pi\left(n+\frac{1}{2}\right)(1-\chi)}{\gamma-1}\right). \tag{S31}$$

Differentiating $\phi_n(\chi, \gamma)$ with respect to $\chi$ and evaluating at $\chi = 1$, we obtain

$$\phi_n'(1,\gamma) = \frac{\pi\left(n+\frac{1}{2}\right)}{\gamma-1}\csc\left(\frac{\pi\left(n+\frac{1}{2}\right)}{\gamma-1}\right). \tag{S32}$$

By evaluating Eq. S17, the coefficients $A_n$ are found to be

$$A_n(\gamma) = \frac{4}{\pi(1+2n)}\sin\left(\frac{\pi\left(n+\frac{1}{2}\right)}{\gamma-1}\right). \tag{S33}$$

Considering only the smallest eigenvalue $\lambda_0$ ($n = 0$), we have

$$A_0(\gamma) = \frac{4}{\pi} \sin\left(\frac{\pi}{2(\gamma - 1)}\right), \qquad \phi_0'(1,\gamma) = \frac{\pi}{2\,(\gamma - 1)} \csc\left(\frac{\pi}{2\,(\gamma - 1)}\right). \tag{S34}$$

Since a plane has no curvature, the concave and convex cases are identical. For simplicity, we consider the case where $\gamma = 0$. Thus, $A_0$ and $\phi_0'$ simplify to

$$A_0(0) = -\frac{4}{\pi}, \qquad \phi_0'(1,0) = \frac{\pi}{2}. \tag{S35}$$

Using Eq. S27, this leads to the final result for the dimensionless diffusion time $\tilde{\tau}$ in a finite plane:

$$\textbf{plane:} \quad \tilde{\tau} = \frac{4 \ln 2}{\pi^2}. \tag{S36}$$

## 7.2 Cylinder

### 7.2.1 Concave cylinder

The eigenvalues for $d = 2$ and $\gamma = 0$ are determined by

$$J_0\left(\lambda_n\right) = 0, \tag{S37}$$

with the corresponding eigenfunctions $\phi_n(\chi, \gamma)$:

$$\phi_n(\chi, 0) = J_0(\lambda_n \chi). \tag{S38}$$

The roots of $J_0$ are transcendental and cannot be expressed in closed form. By evaluating Eq. S17, the coefficients $A_n$ are

$$A_n(0) = \frac{2}{\lambda_n J_1(\lambda_n)}. \tag{S39}$$

Differentiating $\phi_n(\chi, 0)$ with respect to $\chi$ and evaluating at $\chi = 1$, we obtain

$$\phi_n'(1,0) = -\lambda_n J_1(\lambda_n), \tag{S40}$$

and

$$|A_n(0)\phi_n'(1,0)| = 2. \tag{S41}$$

Using Eq. S27, this leads to the final result for the dimensionless diffusion time $\tilde{\tau}$ of a concave cylinder:

$$\textbf{concave cylinder:} \quad \tilde{\tau} = \frac{\ln 2}{\lambda_0^2} \approx 0.173 \ln(2). \tag{S42}$$

### 7.2.2 Convex cylinder

The eigenvalues for $d = 2$ and $\gamma = 2$ are determined by

$$J_0\left(\lambda_n\right) Y_1\left(2\lambda_n\right) - J_1\left(2\lambda_n\right) Y_0\left(\lambda_n\right) = 0, \tag{S43}$$

with the corresponding eigenfuncitons $\phi_n(\chi, \gamma)$:

$$\phi_n(\chi, 2) = \frac{Y_0(\lambda_n \chi) J_0(\lambda_n) - Y_0(\lambda_n) J_0(\lambda_n \chi)}{J_0(\lambda_n)}, \tag{S44}$$

The coefficients $A_n$ are

$$A_n(2) = \frac{\pi J_0(\lambda_n)}{\pi^2 \lambda_n^2 \left[J_0(2\lambda_n) Y_0(\lambda_n) - J_0(\lambda_n) Y_0(2\lambda_n)\right]^2 - 1}. \tag{S45}$$

Differentiating $\phi_n(\chi, 2)$ with respect to $\chi$ and evaluating at $\chi = 1$, we obtain

$$\phi_n'(1, 2) = \frac{2}{\pi J_0(\lambda_n)}, \tag{S46}$$

and

$$|A_n(2)\phi_n'(1, 2)| = \frac{2}{|\pi^2 \lambda_n^2 \left(J_0(2\lambda_n) Y_0(\lambda_n) - J_0(\lambda_n) Y_0(2\lambda_n)\right)^2 - 1|} \tag{S47}$$

The eigenvalues $\lambda_n$ are transcendental, and Eq. S47 must be evaluated numerically. Considering only the smallest eigenvalue $\lambda_0$, we find

$$|A_0(2)\phi_0'(1, 2)| \approx 2.42. \tag{S48}$$

Using Eq. S27, this leads to the final result for the dimensionless diffusion time $\tilde{\tau}$ of a convex cylinder:

$$\textbf{convex cylinder:} \quad \tilde{\tau} \approx \frac{\ln 2.42}{\lambda_0^2} \approx 0.540 \ln(2.42). \tag{S49}$$

### 7.3 Sphere

We begin with the characteristic equation for the eigenvalues considering $d = 3$:

$$\tan\left(\lambda_n(\gamma - 1)\right) = \gamma \lambda_n. \tag{S50}$$

The corresponding eigenfunctions $\phi_n(\chi, \gamma)$ are given by

$$\phi_n(\chi, \gamma) = \frac{\sin\left(\lambda_n(\chi - 1)\right)}{\lambda_n \chi}. \tag{S51}$$

Differentiating $\phi_n(\chi, \gamma)$ with respect to $\chi$ and evaluating at $\chi = 1$, we obtain

$$\phi_n'(1, \gamma) = 1. \tag{S52}$$

By evaluating Eq. S17, the coefficients $A_n$ are found to be

$$A_n(\gamma) = \frac{4\left(\sin\left((\gamma - 1)\lambda_n\right) - \lambda_n\left(1 - \gamma \cos\left((\gamma - 1)\lambda_n\right)\right)\right)}{\sin\left(2(\gamma - 1)\lambda_n\right) - 2(\gamma - 1)\lambda_n}. \tag{S53}$$

#### 7.3.1 Concave sphere

For $\gamma = 0$ we have

$$A_n(0) = \frac{4\left(\lambda_n - \sin\left(\lambda_n\right)\right)}{\sin\left(2\lambda_n\right) - 2\lambda_n}. \tag{S54}$$

The eigenvalues are

$$\lambda_n = n\pi, \quad n \in \mathbb{Z}^+. \tag{S55}$$

Substituting $\lambda_n = n\pi$ into the equation for $A_n(0)$ we find

$$A_n(0) = -2. \tag{S56}$$

Using Eq. S27, this leads to the final result for the dimensionless diffusion time $\tilde{\tau}$ of a concave sphere:

$$\textbf{concave sphere:} \quad \tilde{\tau} = \frac{\ln 2}{\pi^2} \tag{S57}$$

#### 7.3.2 Convex sphere

For $\gamma = 2$ we have

$$A_n(2) = \frac{4\left(\sin\left(\lambda_n\right) - \lambda_n\left(1 - 2\cos\left(\lambda_n\right)\right)\right)}{\sin\left(2\lambda_n\right) - 2\lambda_n}. \tag{S58}$$

The eigenvalues $\lambda_n$ are transcendental and must be calculated numerically. Using the smallest eigenvalue $\lambda_0 = 1.17$ we find

$$|A_0(2)\phi_0'(1,2)| \approx 2.90. \tag{S59}$$

Substitution into Eq. S27 leads to the final result for the dimensionless diffusion time $\tilde{\tau}$ of a convex sphere:

$$\textbf{convex sphere:} \quad \tilde{\tau} \approx \frac{\ln 2.90}{\lambda_0^2} \approx 0.736\ln(2.90). \tag{S60}$$

# 8 Summary

The geometry dependent diffusion time can be more easily obtained from Eq. S27 by realizing that this scales with the smallest eigenvalue $\lambda_0$ for any given geometry. In fact,

$$\tilde{\tau} = \mathcal{O}(\lambda_0^{-2}), \tag{S61}$$

which only depends on the geometry, uniquely defined here by $d$ (planar, cylindrical, or spherical) and $\gamma$ (convex or concave).

| | Dimensionless characteristic diffusion time: $\tilde{\tau} \sim \lambda_0^{-2}$ | Characteristic diffusion time (normalized to a plane: $\tilde{\tau}/\tilde{\tau}_{\text{plane}}$) |
|---|---|---|
| Concave Sphere ($d = 3$, $\gamma = 0$) | $\pi^{-2}$ | 0.250 |
| Concave Cylinder ($d = 2$, $\gamma = 0$) | 0.173 | 0.427 |
| Plane ($d = 1$, $\gamma = 0$ or $\gamma = 2$) | $4\pi^{-2}$ | 1 |
| Convex Cylinder ($d = 2$, $\gamma = 2$) | 0.540 | 1.33 |
| Convex Sphere ($d = 3$, $\gamma = 2$) | 0.736 | 1.82 |

Table S1: Characteristic diffusion times for different geometries in a finite domain of size $\ell$.
